# Loss of TTC17 promotes breast cancer metastasis through RAP1/CDC42 signaling and sensitizes it to rapamycin and paclitaxel

**DOI:** 10.1186/s13578-023-01004-8

**Published:** 2023-03-09

**Authors:** Jingyao Zhang, Fengzhu Guo, Chunxiao Li, Yang Wang, Jinsong Wang, Fangzhou Sun, Yantong Zhou, Fei Ma, Bailin Zhang, Haili Qian

**Affiliations:** 1 State Key Laboratory of Molecular Oncology, National Cancer Center/National Clinical Research Center for Cancer/Cancer Hospital, Chinese Academy of Medical Sciences and Peking Union Medical College, Beijing, 100021 China; 2 Department of Medical Oncology, National Cancer Center/National Clinical Research Center for Cancer/Cancer Hospital, Chinese Academy of Medical Sciences and Peking Union Medical College, Beijing, 100021 China; 3 Department of Breast Surgical Oncology, National Cancer Center/National Clinical Research Center for Cancer/Cancer Hospital, Chinese Academy of Medical Sciences and Peking Union Medical College, Beijing, 100021 China

**Keywords:** TTC17, RAP1/CDC42, Metastasis, Breast cancer, Drug sensitivity

## Abstract

**Background:**

Breast cancer (BC) metastasis is the leading cause of poor prognosis and therapeutic failure. However, the mechanisms underlying cancer metastasis are far from clear.

**Methods:**

We screened candidate genes related to metastasis through genome-wide CRISPR screening and high-throughput sequencing of patients with metastatic BC, followed by a panel of metastatic model assays. The effects of tetratricopeptide repeat domain 17 (TTC17) on migration, invasion, and colony formation ability together with the responses to anticancer drugs were investigated in vitro and in vivo. The mechanism mediated by TTC17 was determined by RNA sequencing, Western blotting, immunohistochemistry, and immunofluorescence. The clinical significance of TTC17 was evaluated using BC tissue samples combined with clinicopathological data.

**Results:**

We identified the loss of TTC17 as a metastasis driver in BC, and its expression was negatively correlated with malignancy and positively correlated with patient prognosis. TTC17 loss in BC cells promoted their migration, invasion, and colony formation capacity in vitro and lung metastasis in vivo. Conversely, overexpressing TTC17 suppressed these aggressive phenotypes. Mechanistically, TTC17 knockdown in BC cells resulted in the activation of the RAP1/CDC42 pathway along with a disordered cytoskeleton in BC cells, and pharmacological blockade of CDC42 abolished the potentiation of motility and invasiveness caused by TTC17 silencing. Research on BC specimens demonstrated reduced TTC17 and increased CDC42 in metastatic tumors and lymph nodes, and low TTC17 expression was linked to more aggressive clinicopathologic characteristics. Through screening the anticancer drug library, the CDC42 inhibitor rapamycin and the microtubule-stabilizing drug paclitaxel showed stronger inhibition of TTC17-silenced BC cells, which was confirmed by more favorable efficacy in BC patients and tumor-bearing mice receiving rapamycin or paclitaxel in the TTC17^Low^ arm.

**Conclusions:**

TTC17 loss is a novel factor promoting BC metastasis, that enhances migration and invasion by activating RAP1/CDC42 signaling and sensitizes BC to rapamycin and paclitaxel, which may improve stratified treatment strategies under the concept of molecular phenotyping-based precision therapy of BC.

**Supplementary Information:**

The online version contains supplementary material available at 10.1186/s13578-023-01004-8.

## Introduction

Breast cancer (BC) is a common malignancy among women and accounts for 11.7% of all newly diagnosed cancer cases according to the global cancer burden data released by the International Agency for Research on Cancer (IARC) of the World Health Organization in 2020 [1]. Despite improvements in therapeutic approaches in recent years, the prognosis of advanced BC is still poor. The five-year survival rate of patients with distant metastatic disease is only 27%, which highlights the need for intensive investigation into the mechanism of cancer metastasis [2].

Tumor metastasis is a multistep process involving tumor cell migration, vascular invasion, and expansion in the microenvironment, which is orchestrated by various gene regulation networks and growth factors [3–5]. As the central events, specific mutations and abnormal expression of some genes are critical driving forces for tumor metastasis [6, 7]. To date, only a few master gene events modulating BC metastasis have been identified and verified in terms of their functions and mechanisms [8, 9]. Therefore, it is urgent to identify novel BC metastasis-modulating events and determine drug-sensitive populations based on their molecular phenotypes.

Tetratricopeptide repeat domain 17 (TTC17) is approximately 136 kb long and located in bands 12–11.2 on the short arm of chromosome 11 (11p12-p11.2), comprising 27 exons. It has been reported to play an integral role in primary ciliogenesis by regulating actin polymerization, and it interacts with C2orf62 [10]. RNAi-mediated silencing of TTC17 significantly impairs cilia formation, which is relevant to cancer biology given that cytoskeletal remodeling controls tumor cell motility and invasion [10, 11]. A previous study also reported that TTC17 mutations result in cholangiocyte cilia malformation and dysfunction, which might be a crucial mechanism underlying biliary atresia pathogenesis [12]. TTC17 also interacts with proteins on the endoplasmic reticulum-Golgi membrane, and knocking down TTC17 altered Golgi polarization and structure [13]. However, its role in cancer development and metastasis has not been explored.

Here, we demonstrated that loss of TTC17 function was a vital metastasis driver in BC and exerted its effects by activating the RAP1/cell division cycle 42 (CDC42) pathway. TTC17 loss in BC contributes to BC metastasis, and BC cells lacking TTC17 expression are more sensitive to rapamycin and paclitaxel treatment. This discovery is clinically relevant and can optimize therapeutic approaches by stratifying BCs according to TTC17 status. The present study is the first report of TTC17 on its cancer-related function and potential treatment value.

## Materials and methods

### Cell culture

Human BC MDA-MB-231 and MCF-7 cell lines, murine breast tumor 4T1 cell lines, and human embryonic kidney HEK293T cell lines were purchased from the American Type Culture Collection (ATCC, USA) and authenticated by short tandem repeat profiling. All of the cells were maintained in high-glucose Dulbecco’s modified Eagle’s medium (DMEM; BIOROC) supplemented with 10% fetal bovine serum (FBS; HyClone) and 1% penicillin–streptomycin (BIOROC) in a humidified atmosphere with 5% carbon dioxide at 37 °C.

### Wound healing assays

To evaluate cell migration ability, MDA-MB-231 and MCF-7 cells were seeded in 6-well plates at a density of 5 × 10^5^ cells/well and cultured until confluent. The monolayer was scratched longitudinally with a sterile pipette tip and rinsed twice to remove the dislodged cells. Fresh medium was added, and the wound region was observed at predetermined time points under a light microscope equipped with a digital camera. The wound area coverage was measured by analyzing the images with ImageJ software (version 1.8.0).

### Cell invasion assays

Transwell membranes were coated with Matrigel (40 μg/mL, Corning) at 37 °C for 1 h. The cells treated as indicated were seeded into the precoated upper chambers at a density of 5 × 10^4^ cells/mL in serum-free medium, and the lower chambers were filled with medium containing 20% FBS as the chemoattractant. After culturing for 24 h at 37 °C, the noninvasive cells on the upper surface were removed with a cotton swab, and the cells that migrated to the lower surface through the membrane were fixed with 4% paraformaldehyde for 10 min. The fixed cells were then stained with crystal violet (Beyotime Biotechnology) for 10 min, washed with tap water, and counted under an inverted microscope (Olympus).

### Colony forming assays

The cells were seeded in 6-well plates at a density of 200 cells/well and cultured for 2 weeks. The medium was replaced every 3 days. The colonies were fixed with 4% paraformaldehyde for 10 min, stained with 4% crystal violet (Beyotime Biotechnology) for 10 min, washed several times with tap water and air-dried. The plates were photographed, and the number of colonies was counted using ImageJ software (version 1.8.0).

### In vivo assays in mouse models

Six- to eight-week-old female BALB/c mice, nude mice, and NOD/SCID mice (n = 5–6/group) were purchased from HFK Bioscience (Beijing, China) and housed in a specific pathogen-free environment. All experiments were approved by the Institutional Animal Care and Use Committee of Cancer Hospital, Chinese Academy of Medical Sciences.

For the lung metastasis assay, each BALB/c-nude mouse or NOD/SCID mouse was injected intravenously via the tail vein with 1 × 10^7^ MDA-MB-231 tumor cells and monitored closely for 13 weeks. The mice were then euthanized, and their lungs were harvested. Metastatic nodules were counted, and the tissues were processed for histological analysis. In another lung metastasis model, we injected 5 × 10^5^ 4T1 murine triple-negative BC cells into the third pair of right mammary fat pads of female BALB/c mice. After 35 days, the mice were sacrificed and subjected to follow-up investigations.

For the subcutaneous tumor model and paclitaxel treatment, 2 × 10^5^ 4T1 cells were implanted into BALB/c mice, and tumor measurements were taken with a caliper every 3 d. Tumor-bearing mice were randomly allocated to receive intraperitoneal injection of paclitaxel (10 mg/kg) or vehicle three times weekly once the tumor volume was approximately 50–100 mm^3^. After two weeks of consecutive administration, the mice were sacrificed, and the tumor masses were resected, photographed, and weighed. Tumor volume was calculated using the following formula: 0.52 × length × width^2^.

### Plasmid constructs

TTC17-specific short hairpin RNA sequences (Additional file 1: Table S1) and the scrambled nontarget sequence were subcloned into the pLKO.1 vector. The recombinant TTC17-overexpressing plasmid was synthesized by amplifying the TTC17 gene using specific primers and cloning the amplicons into the pCDH vector. *E. coli DH5α* cells were transformed with different constructs, and the plasmids were extracted as per standard protocols.

### Recombinant lentivirus construction and infection

HEK293T cells were cultured until 60% confluence in 6 cm plates and cotransfected with 2 μg of the appropriate plasmids and 2.5 μg of helper plasmids (1.5 μg psPAX2 and 1 μg pmD2.G) using Lipofectamine 2000 (Invitrogen) according to the manufacturer’s instructions. The supernatants containing viral particles were collected 48 h and 72 h later and filtered through 0.45 μm polyvinylidene fluoride (PVDF) membranes. MDA-MB-231, MCF-7, and 4T1 cells were transduced with viral supernatants containing 5 μg/mL polybrene, and the medium was replaced 48 h later. The stably transduced cells were selected by culturing in the presence of 2 μg/mL puromycin for at least 7 days. Subsequently, the cells were harvested for Western blotting.

### Genome-scale CRISPR–Cas9 screening

Genome-wide CRISPR–Cas9 knockout (GeCKO) v2.0 library plasmids were kindly provided by Feng Zhang [14]. The GeCKOv2.0 library A consists of 65,383 single guide RNAs (sgRNAs) that target 19,050 genes and 1864 miRNAs, causing frameshift indel mutations that lead to loss-of-function alleles. MCF7 cells were transduced with lentivirus carrying the GeCKOv2.0 library at an MOI of 0.2–0.4 for 24 h to achieve 100 × coverage of each sgRNA construct and then selected with puromycin (3 μg/mL) for 7 days. Puromycin-resistant cells were expanded for another 10 days to allow gene editing. Transwell invasion assays were performed as described, and invasive and noninvasive cells were harvested. Genomic DNA was isolated from the cells using a Blood & Cell Culture Midi kit (Qiagen). The sequences targeted by sgRNAs were amplified by the two-step PCR method, and the primer sequences for lentiCRISPR sgRNAs are listed in Additional file 1: Table S1 [14, 15]. The PCR products were purified by agarose gel electrophoresis, quantified using a Qubit 3.0 Fluorometer (Invitrogen) and an ABI7900 real-time fluorescence quantitative PCR instrument (Applied Biosystems) and sequenced on a HiSeq 2500 instrument (Illumina) in single-end mode. The MAGeCK algorithm was used to analyze the FASTQ files and identify metastasis-related genes.

### Immunoprecipitation

The cells were washed three times in cold PBS. The cold radioimmunoprecipitation assay (RIPA) lysis buffer containing protease inhibitors and phosphatase inhibitors was added to each well. The cells were transferred into 1.5 mL Eppendorf tubes and the lysates were centrifuged by rotating at 4℃ for 10 min at 16000 × g. Immunocoprecipitation was performed by combining cell lysate proteins with 10 μL Dynabeads protein G (Invitrogen, Carlsbad, CA) and mouse anti-RAP1-GTP antibodies (NewEast Bioscience, King of Prussia, PA), followed by incubating overnight and lightly rotating at 4 °C. The complex was washed 6 times with the RIPA cracking buffer, re-suspended in the 2 × loading buffer, and denatured at 95 °C for 5 min.

### Western blotting

Suitably transfected cells were cultured for 48 h and lysed with RIPA buffer supplemented with a proteinase/phosphatase inhibitor cocktail. The lysates were separated by 10% SDS–PAGE and transferred to PVDF membranes. After blocking for 1 h with PBST containing 5% skim milk, the blots were incubated overnight with the appropriate primary antibodies at suitable dilutions. The protein bands were probed with horseradish peroxidase (HRP)-conjugated secondary antibodies, and the positive bands were visualized using Pierce ECL Western blotting Substrate (Thermo Scientific). The density of the bands was analyzed using ImageJ software (version 1.8.0). ACTIN was used as the internal control.

### Immunohistochemistry (IHC)

The paraffin sections were dewaxed, rehydrated, and heated for antigen retrieval. After incubation with the primary antibody for 1 h, the sections were washed and then probed with HRP-conjugated secondary antibody. The slides were imaged on a digital tissue section scanner (Leica Biosystems), and the average signal intensity and proportion of positively stained cells in each field were calculated. Both values were combined to generate semiquantitative H-scores ranging from 0 to 300 [16, 17].

### Tissue sample selection

During the screening stage of candidate genes, we collected tumor tissues from 26 BC patients, divided them into groups without lymph node metastasis and multiple lymph node metastases, and performed whole-exome sequencing (WES). The inclusion criteria for the metastatic group were a longest tumor diameter of 5–28 mm and ≥ 5 lymph node metastases. Cases without lymph node metastasis were defined as those with a maximum tumor diameter of 31–50 mm. Patients with fewer than 10 lymph node dissections were excluded due to insufficient pathological samples as per the current clinical diagnosis and treatment guidelines. In terms of TNM staging, patients in the metastatic arm were required to meet the following criteria: (1) T: T1 or T2; (2) N: N2 or N3; (3) M: M0 or M1. The inclusion criteria for the nonmetastatic arm were as follows: (1) T: T3 or T4; (2) N: N0; (3) M: M0. Patients with other tumor stages or types were excluded. In the validation phase of TTC17 and CDC42, according to the same criteria as above, samples of primary tumors and metastatic lymph node lesions were gathered retrospectively from BC patients with multiple lymph node metastases, along with primary tumor tissues from nonmetastatic BC cases. All formalin-fixed, paraffin-embedded (FFPE) archival samples were obtained from the Department of Pathology and were retrieved after receiving approval from the Ethics Committee of Cancer Hospital, Chinese Academy of Medical Sciences (CHCAMS) along with patient informed consent (NCC2021C-369).

### Clinical data collection

In the current study, 1083 medical records of eligible BC patients who had undergone TTC17 determination at the transcriptome level were obtained from the Cancer Genome Atlas (TCGA, https://portal.gdc.cancer.gov/) dataset. Moreover, 55 medical records of breast cancer patients treated with rapamycin (sirolimus or everolimus) in CHCAMS were collected. We retrospectively evaluated the key demographic features and clinicopathological characteristics, such as patient age, menopausal status, ethnicity, histological type, clinical stage, molecular subtype, and therapeutic management, to assess baseline factors associated with TTC17 expression. Moreover, data for the analysis of gene expression and treatment response were downloaded from ROC plotter (http://www.rocplot.org/) [18]. All information presented in this article was stripped of patient identifiers.

### Drug screening

A total of 313 compounds (approved by the FDA, USP, JP, CFDA, INN, JAN, PMDA, Canada, BAN, EMA, EP, BJP, BP, INN, USAN, Poland) targeting a variety of signaling pathways, including DNA damage, angiogenesis, metabolism, and epigenetics, were tested in two cell lines by the CCK8 assay. Briefly, the cells were seeded in 384-well culture plates at a density of 1000 cells/well in 40 μL media and incubated overnight. Different drugs were added at concentrations of 10, 2, 0.4, 0.08, and 0.016 μM, and 10 μM staurosporine was used as the positive control. After culturing for 72 h, 5 μL CCK8 was added to each well, and the cells were incubated for 1–4 h. The optical density (OD) of the wells was measured at 450 nm, and the inhibition rate of each drug was calculated as (OD_S_-OD_NC_)/(OD_PC_-OD_NC_) × 100%, where OD_S_, OD_NC_, and OD_PC_ correspond to the sample, negative control (DMSO), and positive control, respectively.

### WES analysis

In brief, tumor DNA libraries were prepared from the genomic DNA of macrodissected and FFPE samples using SureSelect Clinical Research Exome kits (Agilent), and paired-end 75 base pair sequencing was performed on the HiSeq 4000 platform (Illumina). The sequencing data were analyzed as described previously, and the reads were aligned to the reference human genome GRCh37 using Burrows–Wheeler Aligner [19, 20]. Duplicate removal, local realignment, and base quality recalibration were performed by the Genome Analysis Toolkit (v3.1.1), and MuTect (v.1.1.4) and Strelka (v3.1.1) were used to identify somatic single nucleotide variants and indels.

### RNA sequencing

Total RNA was isolated from cells treated as indicated using TRIzol reagent (Invitrogen) according to the manufacturer’s protocol and quantified on a NanoDrop (Thermo Fisher Scientific). Whole-transcriptome ribosomal RNA-depleted libraries were generated from 550 ng total RNA by a TruSeq Stranded Total RNA Library Prep Kit with Ribo-Zero Gold High Throughput (Illumina) and sequenced on the Hi-Seq 4000 (Illumina) in the paired-end 75 base pair mode. Adapter sequences and low-quality reads were removed from the Fastq files using Trimmomatic (v0.33), and the filtered reads were mapped to the human reference genome GRCh38.84 using STAR aligner (v2.5.3a). The number of reads per annotated gene was calculated using HTSeq (v0.6.0) following data normalization, and the differentially expressed genes (DEGs) were screened using the limma voom function (v3.32.10) in R language (v3.4.1).

### Bioinformatics analysis

cBioPortal for Cancer Genomics (http://www.cbioportal.org), an open-access online tool containing the raw data of several large-scale genomics projects, was used to identify the coexpressed genes among the candidate genes [21]. The biological functions and signaling pathways associated with these genes were explored with the Database for Annotation Visualization and Integrated Discovery (DAVID, https://david.ncifcrf.gov/) through Kyoto Encyclopedia of Genes and Genomes (KEGG) and Gene Ontology (GO). Data derived from TCGA, Genotype-Tissue Expression (GTEx, https://gtexportal.org/home/), and the UALCAN database (http://ualcan.path.uab.edu/) were used to evaluate the differences in the expression of identified genes between neoplastic and normal tissues in pan-cancer or especially BC based on molecular subtype and clinical stage [22–24]. The expression of TTC17 among BC cell lines was quantified by Cancer Cell Line Encyclopedia (CCLE, https://sites.broadinstitute.org/ccle) [25]. The single-cell RNA-sequencing (scRNA-seq) dataset was available and analyzed in the Single Cell Portal (https://portals.broadinstitute.org/single_cell) [26]. The prognostic value of the important genes was investigated using Kaplan–Meier plotter (https://kmplot.com/analysis/) [27].

### Statistical analysis

The data are expressed as the mean ± SEM of at least three independent experiments. Unpaired Student’s t test and the Wilcoxon rank-sum test were used to compare the continuous variables between two groups with or without normally distributed variables, respectively. The correlation between variables was measured using Pearson’s or Spearman’s correlation coefficient. The association between clinicopathologic factors and TTC17 expression was evaluated using the chi-square test on categorical variables. The effect of TTC17 on clinicopathologic variables was evaluated using logistic regression. The Kaplan–Meier method and log-rank test were used for survival analysis. All statistical tests were two-sided, and *P* < 0.05 was considered statistically significant. SPSS software (v23.0, SPSS Inc.) was used for statistical analysis.

## Results

### TTC17 deficiency is identified as a metastasis driver in BC via high-throughput omics screening

**Fig. 1:**
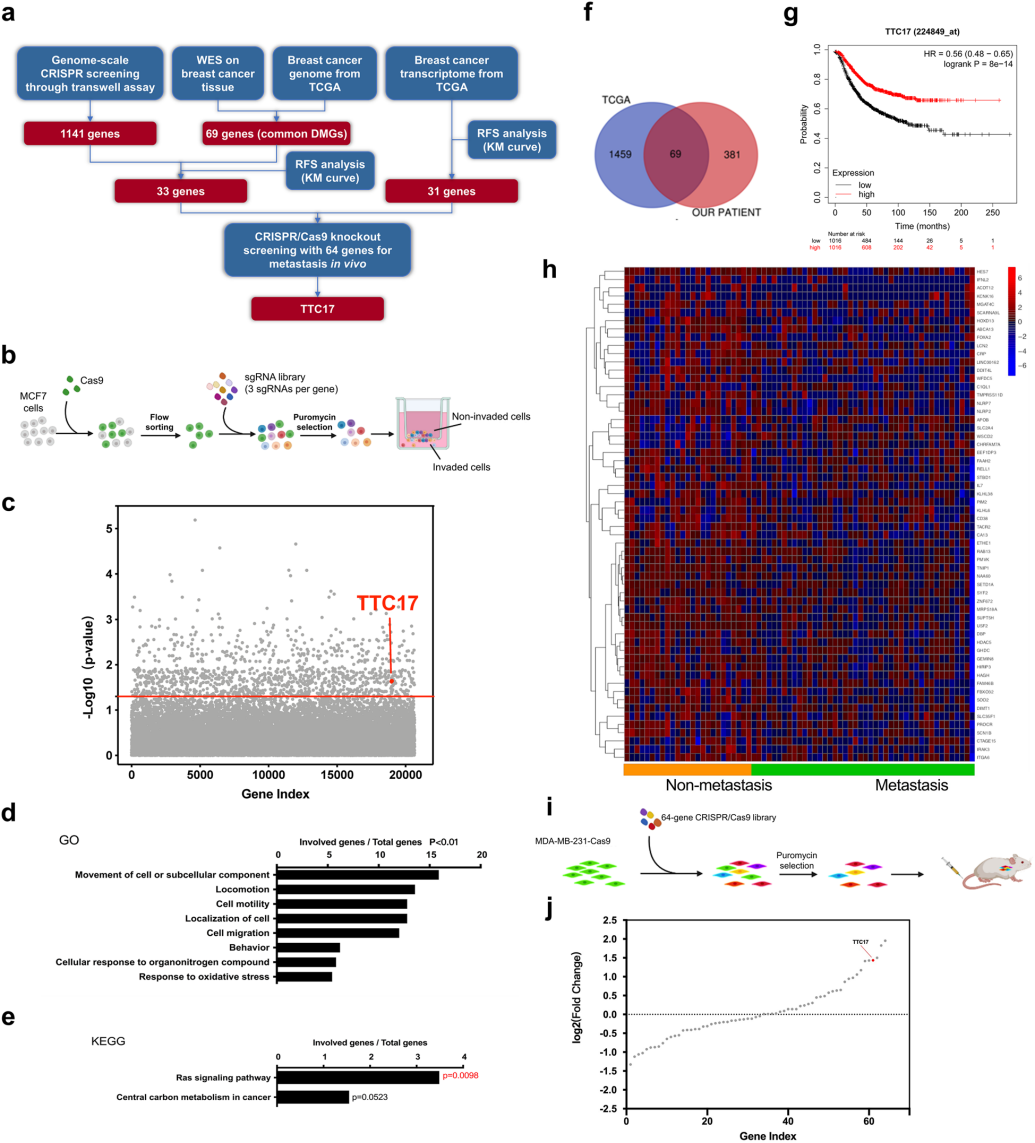
TTC17 deficiency is identified as a metastasis driver in breast cancer via high-throughput omics screening. **a** Flowchart of all screening processes involved in the study. **b** Schematic representation of loss-of-function screening for metastasis-related genes using the human genome-scale CRISPR–Cas9 knockout library linked to Transwell invasion assays. **c** Identification of top genes as candidates concerning their invasion-promoting ability in Transwell screening with MCF7 breast cancer cells using MAGeCK analysis. The red line indicates a *P* value of 0.05. **d, e** GO (**d**) and KEGG (**e**) enrichment for candidate genes identified by GeCKOv.2 library screening in Fig. 1b and c. **f** Overlap of differentially mutated genes between metastatic and nonmetastatic breast cancer from a genomic data comparison in the TCGA database and whole-exome sequencing of tissue samples from our patient cohort. **g** Kaplan‒Meier survival plots of relapse-free survival in breast cancer patients with distinct TTC17 transcriptional expression. **h** Heatmap of representative genes screened by random forest analysis using the transcriptomic data of metastatic and nonmetastatic breast cancer cases from TCGA with low expression promoting metastasis. **i, j** Schematic illustration (**i**) and enrichment of candidate genes (**j**) for a 64-gene CRISPR screening library using in vivo lung metastasis models of MDA-MB-231 breast cancer cells. *CRISPR*, clustered regularly interspaced short palindromic repeats; *WES*, whole-exome sequencing; *TCGA*, the Cancer Genome Atlas; *DMGs*, differentially mutated genes; *RFS*, relapse-free survival; *KM*, Kaplan–Meier; *GO*, Gene Ontology; *KEGG*, Kyoto Encyclopedia of Genes and Genomes; *GeCKO*, genome-scale CRISPR–Cas9 knockout

To obtain reliable candidates connected with BC metastasis, we combined biological assays and data mining to screen metastasis-associated genes. The screening flow is outlined in Fig. 1a. We used the GeCKO v2.0 library, which contains sgRNAs targeting genome-wide genes and miRNAs, to construct gene-edited MCF7 cell lines that were then subjected to puromycin selection and Transwell invasion assays (Fig. 1b). Next-generation sequencing of the DNA extracted from the invaded cells revealed enriched sgRNAs targeting 1141 genes (*P* < 0.05), including TTC17 (Fig. 1c), and functional annotation of the top 286 genes (*P* < 0.01) showed that genes related to cell motility and the Ras signaling pathway were significantly enriched (Fig. 1d, e).

Then, WES of cancer samples from patients eligible for inclusion with metastatic (n = 13) and nonmetastatic (n = 13) BC revealed 450 differentially mutated genes. Further analysis of the genomic data of tumors from 43 metastatic and 25 nonmetastatic patients in the TCGA database revealed 1530 differentially mutated genes. As shown in Fig. 1f, 69 candidate genes, including typical tumor suppressor genes such as TP53 [28–30], CFTR [31–33], LRP5 [34], WWC3 [35–37], and ZNF423 [38, 39], were common to both cohorts. Among them, 58 genes had the same mutation status in the WES and TCGA data, while the remaining genes had different mutation statuses in the two subsets (Fig. 1f, Additional file 1: Table S2). Subsequently, by combining with the results from the genome-wide CRISPR library screen, the high expression profiles of 33 genes were markedly associated with favorable relapse-free survival (RFS; Fig. 1g, Additional file 2: Fig. S1a-h). TTC17 caught our attention because high TTC17 expression had the strongest correlation with improved RFS (hazard ratio 0.56, 95% confidence interval (CI) 0.48–0.65, *P* < 0.001; Fig. 1g, Additional file 2: Fig. S1a-h).

For further confirmation, we also identified the DEGs of primary tumors between the metastatic and nonmetastatic BC cases in the TCGA dataset through random forest analysis combined with survival analysis using the Kaplan–Meier Plotter database, among which the decreased expression of 31 genes was linked to worse RFS (Fig. 1h, Additional file 2: Fig. S1i-p). We next constructed a CRISPR–Cas9 library targeting the 33 genes identified from exome sequencing and the 31 DEGs. Three distinct sgRNAs were designed for each gene, and MDA-MB-231 cells infected with the library were used to establish a lung metastasis model in mice by tail vein injection (Fig. 1i). Eleven weeks later, the genes markedly knocked down in the metastatic lung nodules were identified by sequencing the sgRNA library (Fig. 1j). Of note, we also examined the metastasis of cancer cells to other important sites, such as bone, liver, spleen, kidney, brain, and heart, and found no evidence of metastasis to other organs except the lungs. By summarizing all the above screening results, TTC17 was identified as a metastasis suppressor in BC through genome-scale CRISPR screening, WES and RNA-seq of BC tissues and a mouse model of lung metastasis, and its deletion in BC enhanced metastasis potential. Based on the preliminary experimental results that TTC17 had a more obvious and stable effect on the malignant phenotype of breast cancer than other candidate molecules and our literature review assessing the current status of few studies on TTC17, we finally chose TTC17 for further study.

### TTC17 is downregulated in BC tissue, and low TTC17 expression predicts worse clinical outcomes

**Fig. 2:**
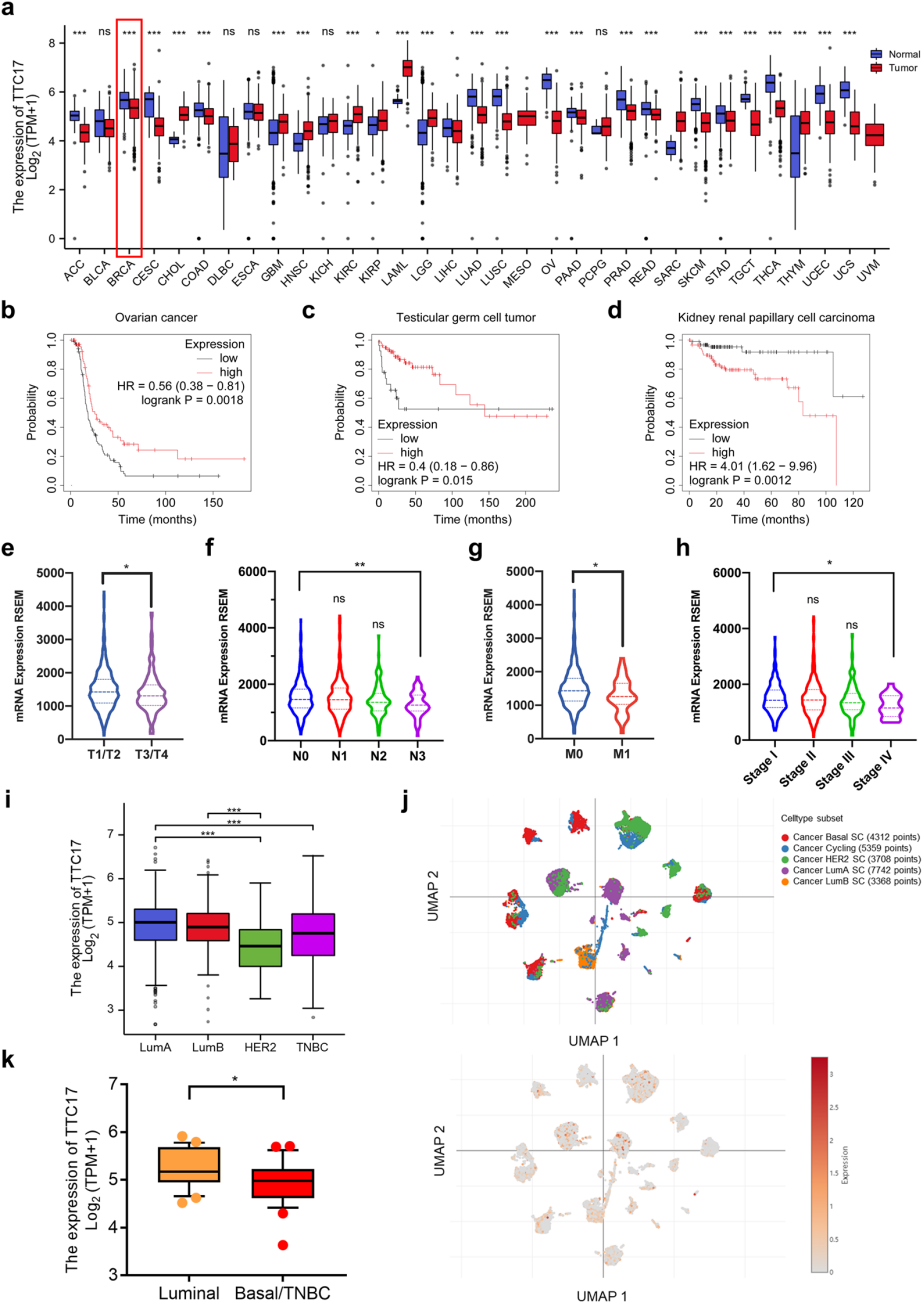
TTC17 is downregulated in breast cancer tissue, and low TTC17 expression predicts worse clinical outcomes. **a** Pancancer analysis of TTC17 expression using data from TCGA combined with the GTEx database. **b–d** Kaplan‒Meier curves of relapse-free survival in patients with ovarian cancer (**b**), testicular germ cell tumor (**c**), or kidney renal papillary cell carcinoma (**d**), stratified by TTC17 expression. Data were obtained from the KM plotter program. **e–i** Association of TTC17 mRNA expression with primary lesion (T stage, **e**), lymph node involvement (N stage, **f**), distant metastasis (M stage, **g**), clinical stage (**h**), or molecular subtype (**i**) in breast cancer patients based on bulk RNA-seq data archived in TCGA. **j** TTC17 expression level in breast cancer with distinct molecular subtypes using single-cell RNA-seq data from the PanglaoDB project. **k** TTC17 expression in luminal or basal/TN breast cancer cell lines based on the CCLE dataset. *P < 0.05, **P < 0.01, ***P < 0.001. *TCGA*, The Cancer Genome Atlas; *GTEx*, Genotype-Tissue Expression; *RNA-seq*, RNA-sequencing; *CCLE*, Cancer Cell Line Encyclopedia

To evaluate the clinical significance of TTC17, we analyzed its transcriptional and translational data using the TCGA, GTEx, UALCAN, and CCLE datasets. Pancancer analysis suggested that TTC17 was mostly downregulated in neoplasms compared to their normal counterparts (Fig. 2a). With regard to prognosis, reduced TTC17 expression was also correlated with shorter RFS in ovarian cancer and testicular germ cell tumors, together with dismal OS in bladder carcinoma, pancreatic ductal adenocarcinoma, rectum adenocarcinoma, and stomach adenocarcinoma (Fig. 2b, c, Additional file 2: Fig. S2a-d). Nevertheless, low TTC17 expression exhibited opposite effects on RFS in kidney renal papillary cell carcinoma and OS in cervical squamous cell carcinoma, kidney renal clear cell carcinoma, kidney renal papillary cell carcinoma, and pheochromocytoma and paraganglioma (Fig. 2d, Additional file 2: Fig. S2e-h).

In BC specifically, TTC17 mRNA and protein expression levels were decreased in the tumor tissues relative to juxta-tumoral tissues (Fig. 2a, Additional file 2: Fig. S3). Moreover, compared to their respective counterparts, breast neoplasms with larger primary lesions, lymph node involvement, distant metastasis, or advanced TNM stage had lower TTC17 expression (Fig. 2e–h). Additionally, the determination of TTC17 based on molecular subtypes indicated that human epidermal growth factor receptor 2 (HER2)-positive and triple-negative BC, representing the intractable subpopulations, had even lower levels of TTC17 expression than luminal subsets with lower aggressiveness (Fig. 2i). scRNA-seq analysis of BC also showed this trend (Fig. 2j). Likewise, TTC17 expression was lower in basal/TNBC cell lines than in luminal BC cell lines (Fig. 2k, Additional file 1: Table S3) [40–42]. Given that higher TTC17 levels were associated with a longer RFS, we surmised that decreased TTC17 expression in breast tissues predisposed individuals to disease progression and metastasis.

### Loss of TTC17 function promotes the metastatic properties of BC cells in vitro and in vivo

**Fig. 3:**
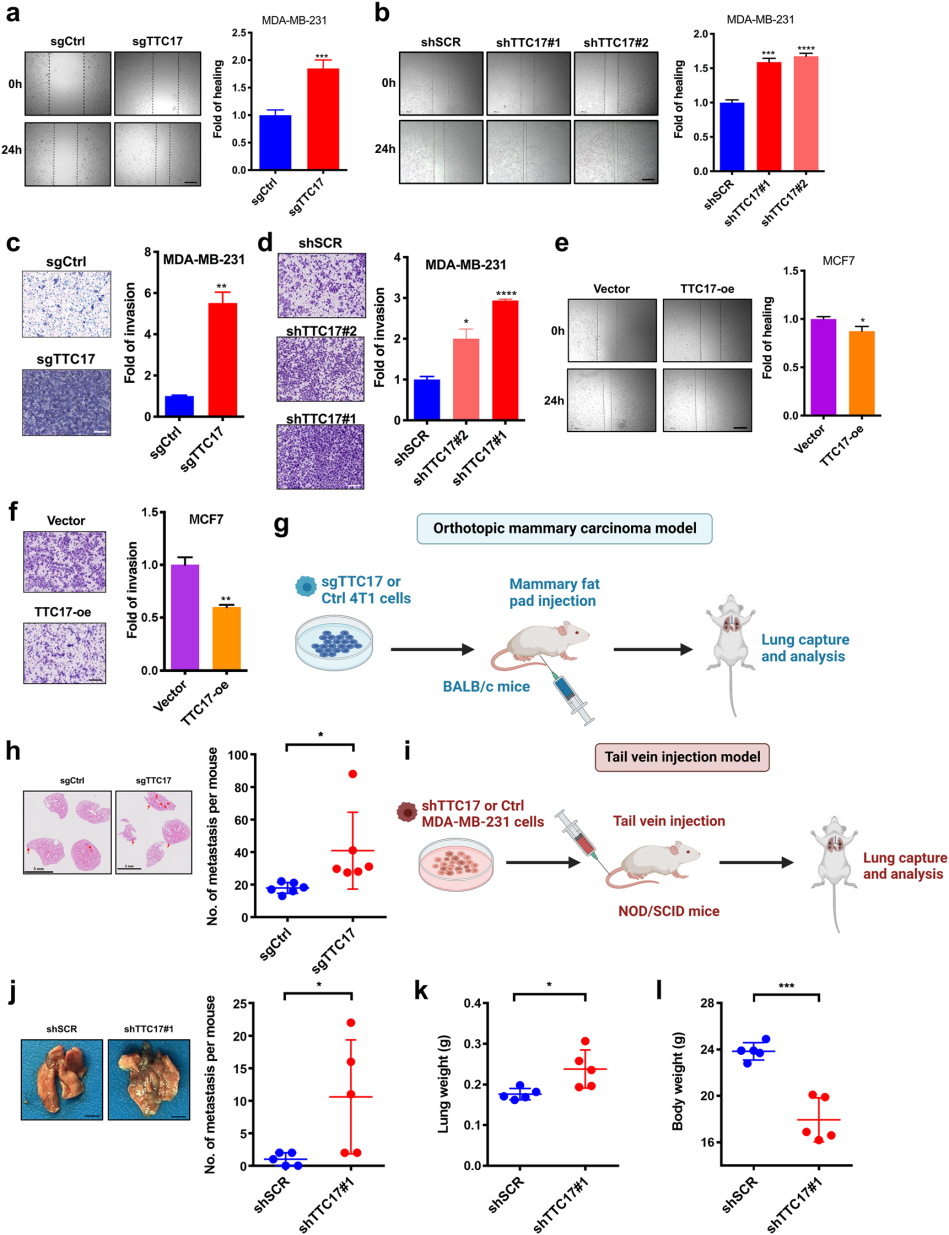
Loss of TTC17 function promotes the metastatic properties of breast cancer cells in vitro and in vivo**. a–d** Representative images and quantitative analysis of wound healing assays (**a**, TTC17 knockout; **b**, TTC17 knockdown) and Transwell invasion assays (**c**, TTC17 knockout; **d**, TTC17 knockdown) using MDA-MB-231 cells with TTC17-deficient and control cells. Scale bars, a, b 500 μm; c-d 200 μm. **e**, **f** Representative images and quantitative analysis of wound healing assays (**e**) and Transwell invasion assays (**f**) using MCF7 cells with TTC17 overexpression and control cells. Scale bars, e 500 μm; f 200 μm. **g** Schematic for evaluating the effect of TTC17 loss on the formation of pulmonary metastasis in the orthotopic mammary carcinoma model. **h** Representative histological images (H&E staining) and quantitative analysis of metastatic nodules in the lungs of BALB/c mice that received 4T1 cells with or without TTC17 depletion. Arrowheads indicate lung metastasis nodules (n = 6 for each group). Scale bar, 5 mm. **i** Schematic for assessing the effect of TTC17 silencing on the initiation of lung colonization through the tail vein injection model. **j–l** Representative gross images and quantitative analysis of metastatic nodules in the lungs (**j**), lung weight (**k**), and bodyweight (**l**) of NOD/SCID mice injected with TTC17-silenced MDA-MB-231 cells and their counterparts (n = 5 for each group). Scale bar, 5 mm. *P < 0.05, **P < 0.01, ***P < 0.001, ****P < 0.0001. *H&E*, hematoxylin and eosin

To determine the biological role of TTC17 in BC, we deleted or silenced TTC17 in MDA-MB-231 and MCF7 cells using the CRISPR–Cas9 system and an shRNA technique. Loss of TTC17 enhanced not only the in vitro migration of MDA-MB-231 cells in wound healing assays but also their invasiveness through Matrigel in Transwell assays (Fig. 3a–d). Forced expression of TTC17 induced the opposite effects (Fig. 3e, f, Additional file 2: Fig. S4). Furthermore, MCF7 cells overexpressing TTC17 formed much smaller and fewer colonies than the controls (Additional file 2: Fig. S5). Thus, TTC17 deficiency promoted the metastatic progression of BC cells.

We next established an orthotopic mouse mammary carcinoma model in female BALB/c mice using TTC17-knockout 4T1 cells to determine the influence of TTC17 on metastatic lung colonization (Fig. 3g). TTC17 depletion substantially accelerated the colonization of BC cells in the lungs, as evaluated by histopathological changes and the number of metastatic nodules (Fig. 3h). Furthermore, we confirmed this property by constructing a lung metastasis model by tail vein injection in NOD/SCID mice using TTC17-silenced MDA-MB-231 cells (Fig. 3i). After 13 weeks, compared to the control group, pronounced increases in metastatic lung nodules and lung weight, in addition to a striking reduction in body weight, were observed in the mice injected intravenously with TTC17-knockdown cancer cells (Fig. 3j-l). Taken together, loss of TTC17 function could boost the metastatic capacity of BC in mouse and human cell-derived models.

### Activation of RAP1/CDC42 signaling is required for TTC17 deficiency to facilitate metastasis

**Fig. 4:**
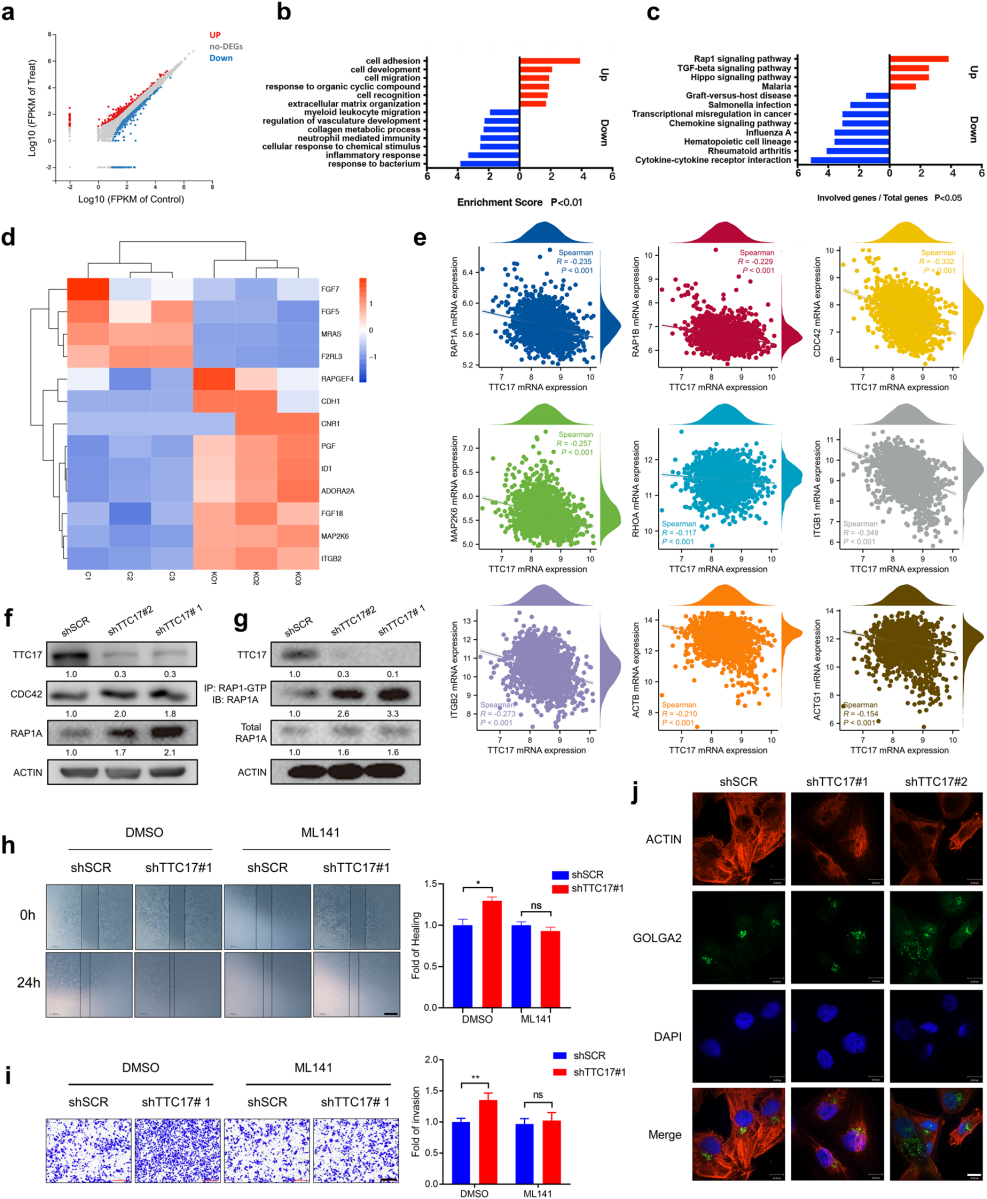
Activation of RAP1/CDC42 signaling is required for TTC17 deficiency to facilitate metastasis. **a** Profile of DEGs (logFC > 1.5, FDR < 0.05) between TTC17-knockout MDA-MB-231 cells and control cells. **b** Enrichment of GO biological processes for up- and downregulated genes. **c** Enrichment of KEGG pathways for up- and downregulated genes. **d** Heatmap of key molecules in the RAP1 signaling pathway enriched by DEGs described in Fig. 4a. **e** mRNA expressional correlation of TTC17 with members of the RAP1/CDC42 cascade in breast cancer, including RAP1A, RAP1B, CDC42, MAP2K6, RHOA, ITGB1, ITGB2, ACTB, and ACTG1. **f, g** Western blot analysis of RAP1/CDC42 signaling activation in MDA-MB-231 cells with or without TTC17 knockdown by increased RAP1A, CDC42 (**f**), and RAP1A-GTP (**g**). **h, i** Representative images and quantitative analysis of wound healing assays (**h**) and Transwell invasion assays (**i**) using TTC17-silenced MDA-MB-231 cells treated with the CDC42 inhibitor ML141 and their counterparts. Scale bars, h 500 μm; i 200 μm. **j** Representative immunofluorescence images of actin cytoskeleton and Golgi morphology in MDA-MB-231 cells with TTC17 silencing or scramble control. Scale bar, 10 μm. *P < 0.05, **P < 0.01, ***P < 0.001. *DEGs*, differentially expressed genes; *FDR*, false discovery rate; *GO*, Gene Ontology; *KEGG*, Kyoto Encyclopedia of Genes and Genomes; *BRCA*, breast invasive carcinoma

Subsequently, we investigated the mechanism by which TTC17 modulated malignant behaviors. Transcriptome sequencing of the control and TTC17-knockout MDA-MB-231 cells revealed DEGs that were notably enriched in biological processes related to metastasis, such as regulation of angiogenesis, cell movement, extracellular matrix, cell adhesion, and cell migration (Fig. 4a, b). In the KEGG pathway enrichment analysis, we observed that the RAP1 signaling pathway was prominently upregulated in TTC17-knockout cells, suggesting its possible involvement in TTC17-mediated BC cell motility and invasiveness (Fig. 4c, d). Multiple RAP1 signaling pathway factors, such as RAP1A, RAP1B, CDC42, MAPK2K6, RHOA, ITGB1, ITGB2, ACTB, and ACTG1, were inversely correlated with TTC17 in the coexpression assessment using the METABRIC dataset, which underscored the role of this pathway in the TTC17-mediated metastatic capacity of BC (Fig. 4e).

Among the RAP1 pathway network members, CDC42 is a GTPase markedly regulated by TTC17. CDC42 was expressed at a higher level in BC than in related normal tissues per the TCGA and GTEx databases (Additional file 2: Fig. S6). TTC17 knockdown in BC cells significantly increased the expression of RAP1A, RAP1-GTP, and CDC42 levels (Fig. 4f, g, Additional file 2: Fig. S7a), while overexpressing TTC17 produced the opposite phenomena (Fig. S7b). These findings suggest that TTC17 silencing activates the RAP1 signaling pathway by releasing RAP1A and CDC42. To validate the contribution of this pathway to cancerous behaviors, we treated TTC17-knockdown BC cell lines with the CDC42 inhibitor ML141. Pharmacological blockade of CDC42 abrogated the compelling difference in migration and invasion ability between MDA-MB-231 cells with TTC17-knockdown shRNA or scramble control, thereby confirming that the TTC17-mediated RAP1/CDC42 pathway was responsible for tumor progression (Fig. 4h, i). Biologically, TTC17 is localized to the Golgi membrane and is required to process cargo proteins and maintain Golgi homeostasis, and its loss leads to Golgi swelling, dilation, and polarization disorders [13]. Consistent with these findings, we also observed dilated and enlarged Golgi in the cells with silenced TTC17, as well as altered actin cytoskeletal organization, as reported previously (Fig. 4j). In conclusion, the RAP1/CDC42 cascade exerted an indispensable effect on the metastatic capability of TTC17 impairment.

### TTC17/RAP1/CDC42 pathway activation correlated with the clinical metastasis and aggressive characteristics of BC

**Fig. 5:**
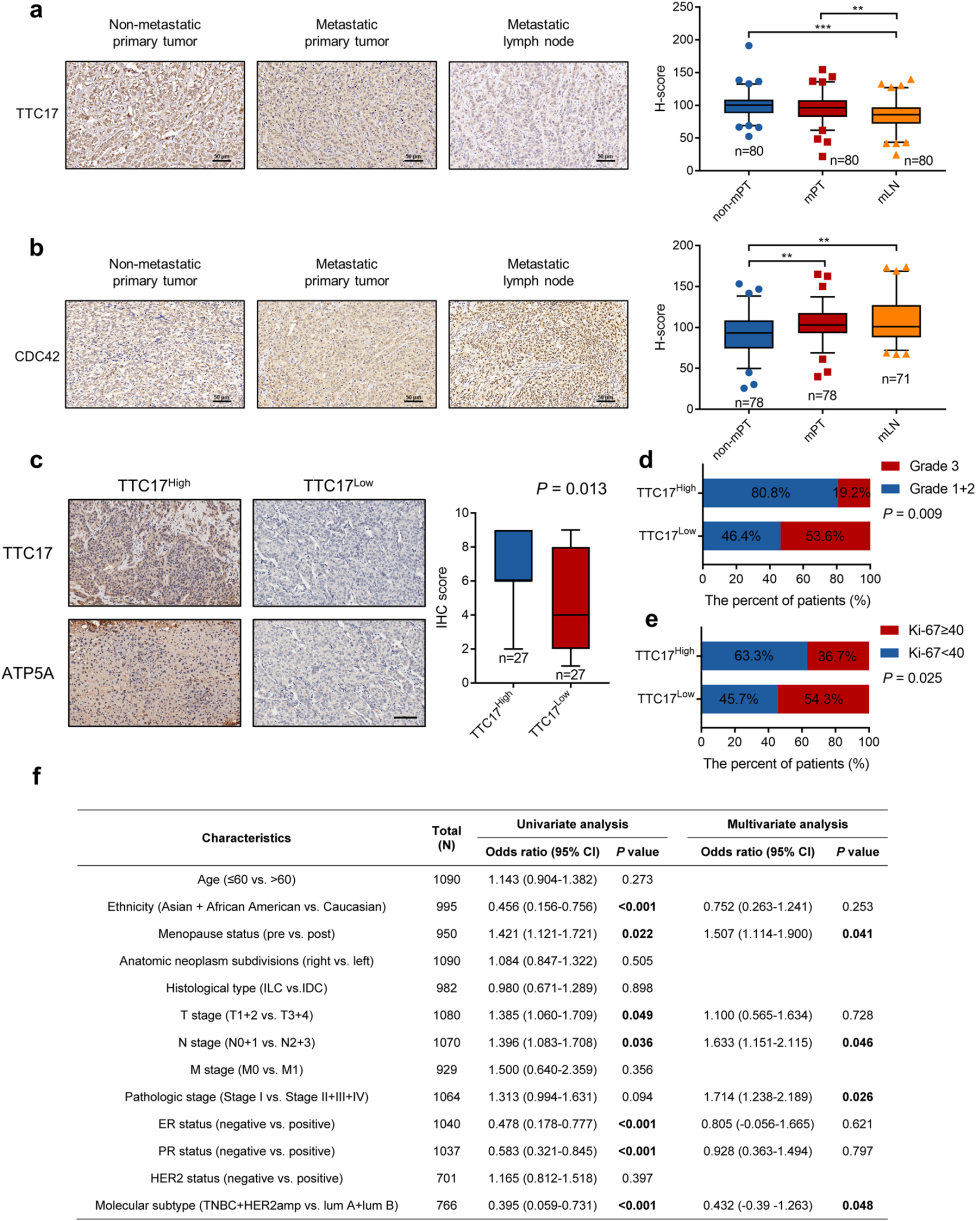
TTC17/RAP1/CDC42 pathway activation correlated with the clinical metastasis and aggressive characteristics of breast cancer. **a, b** Representative images and quantitative analysis of IHC staining of TTC17 (**a**) and the RAP1 signaling pathway effector CDC42 (**b**) in the primary tumor and metastatic lymph node tissues of breast cancer patients. Scale bars, a 50 μm, b 50 μm. **c** Representative IHC images and quantitative analysis of ATP5A expression in patients with breast cancer. Scale bar, 100 μm. **d, e** Correlation between the expression of TTC17 and tumor histopathologic grade (**d**) together with the Ki-67 index (**e**) in our breast cancer cohort. **f** Univariate and multivariate logistic regression analysis of the linkage between TTC17 expression and clinicopathological features in breast cancer patients using data archived in TCGA. Bold text represents statistically significant difference. *IHC*, immunohistochemistry; *non-mPT*, non-metastatic primary tumor; *mPT*, metastatic primary tumor; *mLN*, metastatic lymph node; *TCGA*, The Cancer Genome Atlas *CI*, confidence interval; *ILC*, infiltrating lobular carcinoma; *IDC*, infiltrating ductal carcinoma; *ER*, estrogen receptor; *PR*, progesterone receptor; *HER2*, human epidermal growth factor receptor 2; *TNBC*, triple-negative breast cancer; *lum A*, luminal A; *lum B*, luminal B

To address the contribution of TTC17/RAP1/CDC42 activation to metastasis in patients with BC, we detected the protein expression of TTC17 and CDC42 in a large cohort of nonmetastatic and metastatic primary breast tumors together with their metastatic lymph node tissues. Overall, the primary tumor lesions exhibited higher H-scores (evaluation of IHC staining) for TTC17 than the metastatic lymph nodes, whereas CDC42 expression demonstrated the opposite trend: nonmetastatic primary lesions displayed substantially lower CDC42 expression than metastatic tumors and corresponding metastatic lymph nodes (Fig. 5a, b). Meanwhile, data from another of our BC cohorts suggested that the TTC17^Low^ arm had significantly reduced expression of ATP5A, a molecule that has been reported to have an inverse correlation with metastasis (Fig. 5c) [43].

Moreover, we observed that low expression of TTC17 resulted in higher susceptibility to a higher histopathologic grade of foci as well as a Ki-67 index ≥ 40 than high TTC17 expression (grade 3: 53.6% for TTC17^Low^, 19.2% for TTC17^High^, *P* = 0.009; Ki-67 ≥ 40: 54.3% for TTC17^Low^, 36.7% for TTC17^High^, *P* = 0.025; Fig. 5d, e). Furthermore, we divided the TCGA BC cohort into two groups based on their high or low expression of TTC17. Univariate logistic regression analysis demonstrated that low TTC17 expression was more common in Asians or African Americans *versus* Caucasians (odds ratio (OR) 0.456, 95% CI 0.156–0.756, *P* < 0.001), postmenopausal *versus* premenopausal women (OR 1.421, 95% CI 1.121–1.721, *P* = 0.022), patients with T stage 3 or 4 *versus* 1 or 2 (OR 1.385, 95% CI 1.060–1.709, *P* = 0.049), patients with N stage 2 or 3 *versus* stage 0 or 1 (OR 1.396, 95% CI 1.083–1.708, *P* = 0.036), patients with negative *versus* positive ER status (OR 0.478, 95% CI 0.178–0.777, *P* < 0.001), patients with negative *versus* positive PR status (OR 0.583, 95% CI 0.321–0.845, *P* < 0.001), and patients with TNBC or HER2amp subtype *versus* luminal subtype (OR 0.395, 95% CI 0.059–0.731, *P* < 0.001). Subsequent multivariate logistic regression analysis revealed that postmenopausal *versus* premenopausal status (OR 1.507, 95% CI 1.114–1.900, *P* = 0.041), N stage 2 or 3 *versus* stage 0 or 1 (OR 1.633, 95% CI 1.151–2.115, *P* = 0.046), pathologic stage II, III or IV *versus* stage I (OR 1.714, 95% CI 1.238–2.189, *P* = 0.026), and TNBC or HER2amp subtype *versus* luminal subtype (OR 0.432, 95% CI − 0.39–1.263, *P* = 0.048) were independent factors associated with the low expression of TTC17 (Fig. 5f, the threshold for inclusion in the multivariate analysis after the univariate analysis was controlled for a *P* value < 0.1). The association between the expression of TTC17 and clinicopathological characteristics regarding aggressive malignancy and a poor prognosis further verified its role in facilitating BC progression.

### Loss of TTC17 sensitizes BC to rapamycin and paclitaxel

**Fig. 6:**
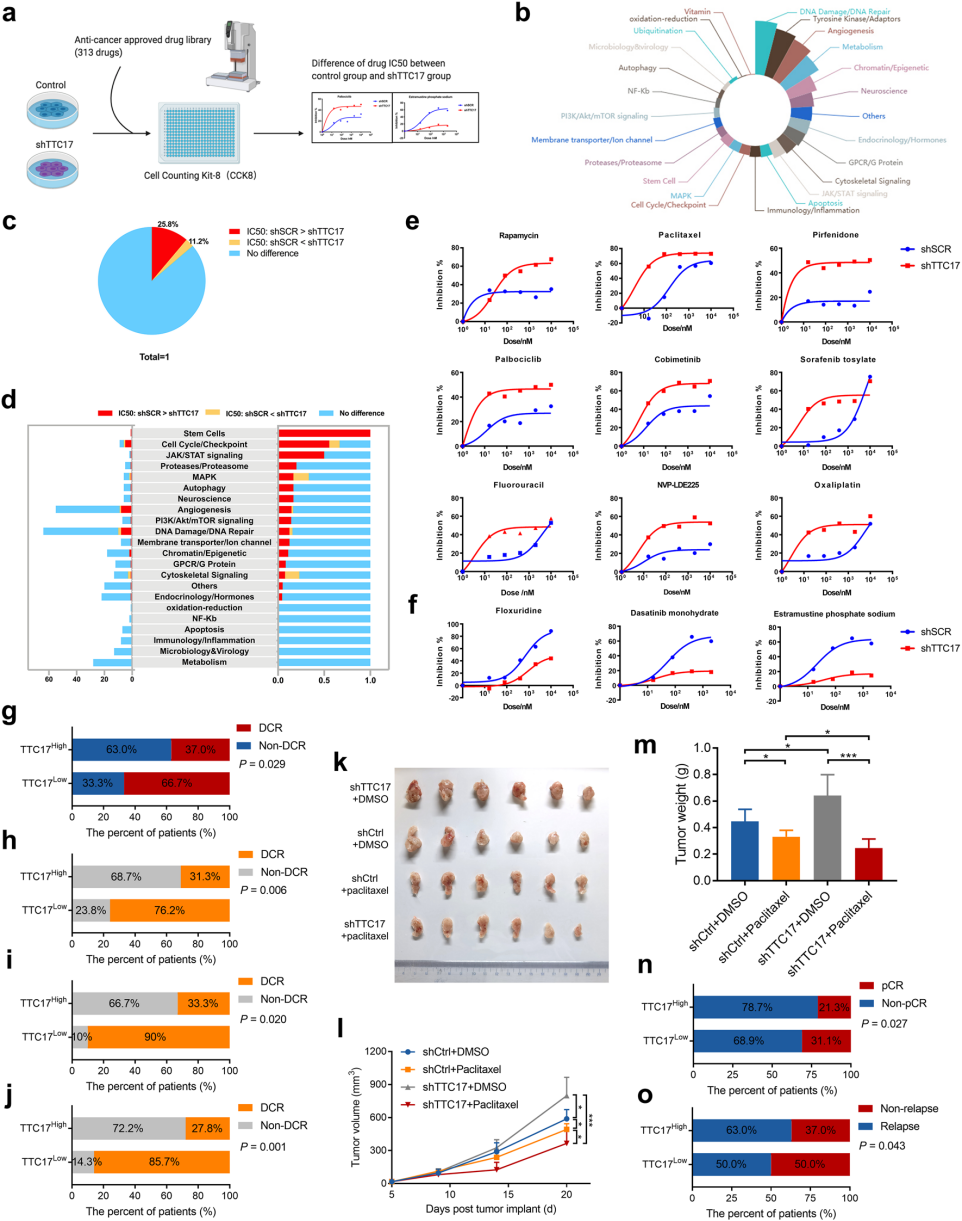
Loss of TTC17 sensitizes breast cancer to rapamycin and paclitaxel. **a** Schematic showing the screening of drugs with a discrepancy in the efficacy on breast cancer cells by TTC17 silencing in the listed antineoplastics through CCK8 assays. **b** Rose plot of the composition of the signaling pathways targeted by the compounds being tested. **c–d** Proportions (**c**) and profiles of signaling networks (**d**) of drugs with inhibitory disparity for TTC17-knockdown and control cells. **e–f** Representative agents, whose inhibitory effects on breast cancer cells were enhanced (**e**) or attenuated (**f**) by TTC17 silencing, including rapamycin and paclitaxel. **g-j** Disease control rates of the breast cancer patients treated with rapamycin in the overall cohort (**g**), in subgroups with patients < 60 years of age (**h**), with pathological grade 1 or 2 (**i**), or with a combination therapy of nonsteroidal aromatase inhibitor or exemestane (**j**). **k–m** Gross image (**k**) and statistical analysis of volume (**l**) and weight (**m**) of the transplanted tumors extracted from BALB/c mice injected with 4T1 cells. **n–o** Association between TTC17 expression and pathologic complete response in breast cancer patients subjected to paclitaxel-based chemotherapy as a neoadjuvant therapy (**n**) or relapse-free survival at five years in breast cancer patients receiving paclitaxel-based chemotherapy as an adjuvant therapy (**o**)

To determine whether TTC17 affects the response of BC cells to antineoplastic drugs, we screened an anticancer drug library consisting of 313 commercially available drugs in control and TTC17-knockdown MDA-MB-231 cells (Fig. 6a, b). Compared to controls, cells losing TTC17 showed increased sensitivity to 25.8% of the tested drugs (lower IC50) and decreased sensitivity to 11.2% of the drugs (higher IC50), which acted on different signaling pathway targets (Fig. 6c, d). Specifically, TTC17 deficiency enhanced the intrinsic sensitivity of cancer cells to rapamycin, paclitaxel, pirfenidone, palbociclib, cobimetinib, sorafenib tosylate, fluorouracil, NVP-LDE225, and oxaliplatin and diminished the sensitivity of cells to floxuridine, dasatinib monohydrate, and estramustine phosphate sodium (Fig. 6e).

Notably, rapamycin is an inhibitor of CDC42 and has been applied to treat patients with advanced hormone receptor-positive, HER2-negative BC. A higher disease control rate (DCR) was achieved in the TTC17^Low^ arm of BC patients receiving rapamycin (*P* = 0.029), and stratified analysis demonstrated that in patients aged < 60 years (*P* = 0.006), with pathological grade 1 or 2 (*P* = 0.020), and a combination regimen of nonsteroidal aromatase inhibitor or exemestane (*P* = 0.001), low expression of TTC17 exhibited a more pronounced correlation with higher DCR (Fig. 6g–j). Furthermore, considering the regulatory role of TTC17 on the actin cytoskeleton, we wondered whether TTC17 expression would affect the efficacy of the anti-microtubule chemotherapeutic agent paclitaxel, which is a common chemotherapeutic drug in routine BC treatment and was also indicated by drug library screening in BC. Paclitaxel treatment significantly reversed the accelerated BC cell growth caused by TTC17 knockdown in mice, showing better efficacy in TTC17 low-expressing BC (Fig. 6k–m). The data concerning therapy responses suggested that reduced TTC17 conferred better pathological complete response (pCR) and five-year RFS rates in BC patients subjected to paclitaxel-based chemotherapy in the neoadjuvant and adjuvant settings, respectively (Fig. 6n, o). Taken together, decreased TTC17 levels in BC promoted metastatic behaviors but sensitized the cancers to a panel of routine anticancer drugs, such as rapamycin and paclitaxel, as verified, providing a treatment opportunity for patients with TTC17-related molecular subtypes (Fig. S8). Drugs losing sensitivity to BC cells with low TTC17 expression are suggested to be avoided in future use in TTC17-deficient breast cancers.

## Discussion

Most cancer patient deaths arise from metastasis. As a consequence, a better understanding of the molecular mechanisms underlying cancer metastasis is pivotal to developing effective therapeutic and preventative approaches. Here, we identified loss of TTC17 as a metastasis driver in BC. Reduced TTC17 expression in BC was correlated with more malignancy and a worse prognosis of patients than intact TTC17 expression. Knocking down TTC17 repressed the migration, invasion, and proliferation of BC cells in vitro and in vivo, whereas forced overexpression of TTC17 disrupted the malignant phenotypes. Clues extracted from transcriptome sequencing pointed out that TTC17 affected metastasis-related behaviors via the RAP1/CDC42 signaling cascade. It was confirmed by the upregulation of key molecules representing the pathway in breast cancer cells and clinical patient tissues, and the consequences of pharmacological blockade of the pathway. In addition, the effect of TTC17 on the cytoskeleton was investigated. TTC17 deficiency was found to be associated with more aggressive clinicopathological features and conferred BC more sensitivity to a range of antineoplastics, including rapamycin and paclitaxel.

Strikingly, in Fig. 5a, b, metastatic primary tumor (mPT) had similar TTC17 expression as compared to non-mPT, but its CDC42 expression is upregulated. We believe that there are several reasons for this phenomenon. First, TTC17 may function throughout the metastatic process of breast cancer (tumor cells break through the basement membrane into the lymphatic and blood circulation and finally attach to distant sites and form new lesions), and its expression changes to gradually decrease, so its expression is less reduced in the mPT. Second, the quantitative statistical plots corresponding to immunohistochemical staining showed that the difference in TTC17 expression in mPT compared with non-mPT, although not reaching statistical significance, had a smaller mean value and showed an overall trend of reduced expression. CDC42 is located in the downstream pathway where TTC17 plays a biological role, so the cascade effect of the signaling further amplifies the weak difference in TTC17, resulting in a statistically significant difference in CDC42, that is, mPT has significantly increased CDC42 expression compared with non-mPT. Moreover, CDC42, as an oncogene, is perhaps not regulated by TTC17 alone but also comanipulated by other molecules, such as MAP kinase, ARHGAP31, ARHGAP17 [44–46], due to signaling networks and crosstalk between different pathways.

To date, research on TTC17 regarding its value in cancer biology and treatment is limited, and to our knowledge, the close association of TTC17 and BC is revealed here for the first time. Alshaker et al*.* demonstrated that proto-oncogenic isozyme sphingosine kinase 1 depletion in prostate and BC cells led to the upregulation of TTC17, recognized as one of the cellular “compensatory” responses, and it potentially influences sphingosine kinase inhibitor efficacy [47]. An epigenome-wide association study mapped a differentially methylated CpG site on TTC17 affected by tea consumption in women, and it was linked to a lower risk of cancer [48]. In line with these results, our study demonstrated the protumor role of TTC17 impairment in BC.

We observed decreased TTC17 expression in BC with a high degree of biological malignancy and higher clinical stage, and vice versa; patients with low TTC17 levels harbored recognized markers representing dismal disease outcomes. This indicated that TTC17 might be a potent and promising biomarker with potential clinical applications. In the pancancer analysis, the expression of TTC17 in most cancerous tissues was markedly lower than that in normal tissues. Additionally, high TTC17 expression foreshadows improved RFS of BC, ovarian cancer, and testicular germ cell tumors, suggesting the special role of TTC17 dysfunction in the initiation and progression of these neoplasms involving reproduction. TTC17 deficiency in tumors possesses similar genetic features, carcinogenic properties, and clinicopathological characteristics to some genetic variations, such as BRCA1/2 mutations, with a higher possibility of robust invasiveness, high histological grade, lymph node involvement, relapse, and a worse prognosis, but this speculation demands further experimental verification [49].

The RAP1 signaling pathway regulates multiple biological processes, including cancer metastasis, and the GTPase RAP1A affects cell adhesion, intercellular junctions, and cell polarity [50–52]. A recent study showed that RAP1A is overexpressed in breast tumors, especially in ductal carcinoma and invasive tissues, compared to normal breast tissues. Furthermore, the metastatic cell lines MDA-MB-231 and Hs578t express significantly higher levels of RAP1A than the weakly invasive cell line MCF-7 and the normal breast epithelial cell line MCF-10A [53]. RAP1 promotes the metastatic potential of MCF-7 cells, which can be markedly suppressed by the pharmacological inhibition of RAP1 [54]. CDC42, a Rho GTPase downstream of RAP1A, plays crucial roles in actin polymerization, cell polarization, and metastasis and is the effector of GOLGA2 in BC cells [55, 56]. We found that the CDC42 inhibitor ML141 partially reversed the elevated mobility resulting from the loss of TTC17 function. Thus, these findings provide evidence that TTC17 inhibits BC metastasis by targeting the RAP1/CDC42 signaling pathway.

Rapamycin blocks the activity of small GTPases such as CDC42, RhoA, and Rac1 without affecting their expression, which restrains cytoskeleton reorganization, invadopodia formation, and cell motility [57, 58]. The IC50 of rapamycin was markedly lower in TTC17-deficient BC cells than in control BC cells, which is not surprising because of the inhibitory effect of TTC17 on RAP1/CDC42 signaling. However, the onco-lncRNA Linc-ROR decreased the effect of rapamycin on BC cell migration, invasion, and survival by acting as a ceRNA sponge for miR-194-3p [59]. Another study showed that the antidiabetic drug acarbose improved rapamycin efficacy in metastatic kidney cancer, and the combination treatment substantially reduced lung metastasis in a mouse model [60]. Rapamycin is currently approved for treating BC, and as per our results, patients with low TTC17 expression may benefit more from rapamycin therapy.

Paclitaxel, a widely used antineoplastic agent, is a cyclodecane derived from the bark of the Pacific yew tree. It has emerged as an essential cytotoxic drug for the treatment of breast and ovarian cancers since it stabilizes microtubules in the polymerized form, causing cell death [61]. As already described, TTC17 is involved in ciliogenesis and actin organization, and we observed that paclitaxel exhibited a more substantial inhibitory effect on BC cells with TTC17 knockdown, which might be related to the cytoskeletal signaling of paclitaxel. Moreover, among BC patients receiving paclitaxel-containing regimens as neoadjuvant or adjuvant chemotherapy, low TTC17 expression was positively associated with improved five-year RFS or preoperative pCR, further indicating the influence of TTC17 deficiency on the efficacy of paclitaxel. Considering that paclitaxel is usually administered as a single agent or in combination in (neo)adjuvant and salvage chemotherapy against BC, making it the mainstay of therapeutic regimens, TTC17 expression could be used to identify patients who respond better to paclitaxel in clinical practice.

There are several limitations in our study that should be considered. The molecular mechanisms underlying TTC17-mediated regulation of the RAP1/CDC42 pathway were not explored in every detail and will therefore be the focus of subsequent studies. In addition, the role of TTC17 in different subtypes of BC also warrants further investigation. In the present study, we examined the difference in efficacy of rapamycin and paclitaxel on in vitro and in vivo models with or without TTC17 knockdown, and plan to investigate the influence of targeting mTOR and microtubules on the metastasis of TTC17-downregulated breast cancer in a follow-up study to clarify its linkage with the molecular network regulation of drug responsiveness. Nevertheless, our findings provide novel insights into the mechanisms underlying BC metastasis as well as potential therapeutic optimization.

## Conclusion

To summarize our findings, we found that TTC17 functional impairment contributed to BC metastasis and regulated metastasis-related phenotypes through RAP1/CDC42 signaling. TTC17 deficiency was correlated with more aggressive clinicopathologic characteristics in BC patients and sensitized BC to rapamycin and paclitaxel, thereby acting as a robust predictive and prognostic biomarker for BC. This provided new insights into the mechanism of cancer metastasis and treatment optimization under a molecular phenotyping-based concept.

## Supplementary Information

**Additional file 1: Table S1** Sequences of shRNA for TTC17 knockdown and primers targeting lentiCRISPR sgRNAs. **Table S2** Mutation profiles of 69 differentially mutated genes common to the TCGA and WES subsets. Abbreviations: TCGA, The Cancer Genome Atlas; WES, whole exon sequencing; DEL, deletion; SNP, single nucleotide polymorphism; INS, insertion. **Table S3** Luminal and basal/TNBC cell lines and their TTC17 expression levels from the CCLE database. Abbreviations: TNBC, triple-negative breast cancer; CCLE, Cancer Cell Line Encyclopedia.

** Additional file 2: Fig. S1** **a-p** Representative Kaplan–Meier curves of relapse-free survival in patients with breast cancer, stratified by the expression of candidate genes, based on the KM plotter database. The candidate genes were obtained from genome-wide CRISPR screening concurrent with mutation detection in breast cancer tissues **(a-h)** or differential transcriptional expression in breast cancer using random forest analysis with TCGA data **(i-p)**. Abbreviation: TCGA, the Cancer Genome Atlas. **Fig. S2 a-h **Kaplan-Meier curves of overall survival in patients with bladder carcinoma (**a**), pancreatic ductal adenocarcinoma (**b**), rectum adenocarcinoma (**c**), stomach adenocarcinoma (**d**), cervical squamous cell carcinoma (**e**), kidney renal clear cell carcinoma (**f**), kidney renal papillary cell carcinoma (**g**), or pheochromocytoma and paraganglioma (**h**), stratified by TTC17 expression. Data was obtained from the KM plotter program. **Fig. S3 **Protein levels of TTC17 in normal and breast cancer tissues based on the CPTAC dataset from the UALCAN portal. Abbreviations: CPTAC, Clinical Proteomic Tumor Analysis Consortium; UALCAN, The University of Alabama at Birmingham Cancer Data Analysis Portal. **Fig. S4** Representative images and quantitative analysis of wound healing assays using MDA-MB-231 cells with forced TTC17 expression and control cells. Scale bar, 500 μm. **Fig. S5 **Graphic display and statistic efficiencies of the colonies formed by MCF7 cells with or without forced TTC17 expression. **Fig. S6** Difference in CDC42 expression between BRCA and normal breast specimens using TCGA combined with GTEx data. Abbreviations: BRCA, breast invasive carcinoma; TCGA, The Cancer Genome Atlas; GTEx, Genotype-Tissue Expression. **Fig. S7 a-b **Western blot analysis of TTC17 and CDC42 expression in MCF7 cells with TTC17 knockdown (**a**) or overexpression (**b**) and their counterparts. **Fig. S8 **Illustration of the role and mechanism of TTC17 on promoting breast cancer metastasis and drug sensitivity via RAP1/CDC42 signaling pathway.

## Data Availability

The datasets used and analyzed during the current study are available from the corresponding author on reasonable request.

## Abbreviations

BC: Breast cancer
IARC: International Agency for Research on Cancer
TTC17: Tetratricopeptide repeat domain 17
CDC42: Cell division cycle 42
ATCC: American Type Culture Collection
DMEM: Dulbecco’s modified Eagle’s medium
FBS: Fetal bovine serum
PVDF: Polyvinylidene fluoride
GeCKO: Genome-wide CRISPR–Cas9 knockout
sgRNAs: Single guide RNAs
HRP: Horseradish peroxidase
IHC: Immunohistochemistry
FFPE: Formalin-fixed, paraffin-embedded
CHCAMS: Cancer Hospital, Chinese Academy of Medical Sciences
TCGA: The Cancer Genome Atlas
OD: Optical density
WES: Whole exome sequencing
DEGs: Differentially expressed genes
DAVID: Database for Annotation Visualization and Integrated Discovery
KEGG: Kyoto Encyclopedia of Genes and Genomes
GO: Gene Ontology
GTEx: Genotype-Tissue Expression
CCLE: Cancer Cell Line Encyclopedia
scRNA-seq: Single-cell RNA-sequencing
RFS: Relapse-free survival
HER2: Human epidermal growth factor receptor 2
OR: Odds ratio
CI: Confidence interval
DCR: Disease control rate
pCR: Pathologic complete response
mPT: Metastatic primary tumor
