# Supplementary material for: Loss of TTC17 promotes breast cancer metastasis through RAP1/CDC42 signaling and sensitizes it to rapamycin and paclitaxel
Source: Cell Biosci. 2023 Mar 9;13:50. doi: 10.1186/s13578-023-01004-8 (PMC9996991; doi:10.1186/s13578-023-01004-8)
Supplement: Supplementary file 1 — Additional file 1: Table S1 Sequences of shRNA for TTC17 knockdown and primers targeting lentiCRISPR sgRNAs. Table S2 Mutation profiles of 69 differentially mutated genes common to the TCGA and WES subsets. Abbreviations: TCGA, The Cancer Genome Atlas; WES, whole exon sequencing; DEL, deletion; SNP, single nucleotide polymorphism; INS, insertion. Table S3 Luminal and basal/TNBC cell lines and their TTC17 expression levels from the CCLE database. Abbreviations: TNBC, triple-negative breast cancer; CCLE, Cancer Cell Line Encyclopedia. [file 13578_2023_1004_MOESM1_ESM.docx]

**Table S1** Sequences of shRNA for TTC17 knockdown and primers targeting lentiCRISPR sgRNAs.

| **Category** | **Name** | **Sequence** |
| --- | --- | --- |
| shRNA sequences for TTC17 knockdown | shTTC17#1-F | CCGGCTAGAACTTCCATATAGTATACTCGAGTATACTATATGGAAGTTCTAGTTTTTG |
|  | shTTC17#1-R | AATTCAAAAACTAGAACTTCCATATAGTATACTCGAGTATACTATATGGAAGTTCTAG |
|  | shTTC17#2-F | CCGGGCAGGGACTCTGATGCATATACTCGAGTATATGCATCAGAGTCCCTGCTTTTTG |
|  | shTTC17#2-R | AATTCAAAAAGCAGGGACTCTGATGCATATACTCGAGTATATGCATCAGAGTCCCTGC |
| Primers for lentiCRISPR sgRNAs | F1 | TAAGTAGAGGCTTTATATATCTTGTGGAAAGGACGAAACACC |
|  | F2 | ATCATGCTTAGCTTTATATATCTTGTGGAAAGGACGAAACACC |
|  | F3 | GATGCACATCTGCTTTATATATCTTGTGGAAAGGACGAAACACC |
|  | R | CCGACTCGGTGCCACTTTTTCAA |

**Table S2** Mutation profiles of 69 differentially mutated genes common to the TCGA and WES subsets.

| **Gene** | **Mutation in TCGA** | **Mutation in WES** |
| --- | --- | --- |
| GOLGA2 | DEL, SNP | DEL |
| ANO8 | SNP | SNP |
| TTC17 | SNP | SNP |
| NRDE2 | SNP | SNP |
| MAP6 | SNP | SNP |
| CDH4 | SNP | SNP |
| AGAP4 | SNP | SNP |
| RHBDF1 | SNP | SNP |
| GRIK2 | SNP | SNP |
| KDM6B | DEL | SNP |
| HYDIN | SNP | SNP |
| LOXHD1 | SNP | SNP |
| SH3TC1 | SNP | SNP |
| SYT4 | SNP | SNP |
| INSR | SNP | SNP |
| BRWD3 | SNP | SNP |
| KIAA1324L | SNP | SNP |
| TP53 | DEL, SNP, INS | DEL, SNP |
| LAIR2 | SNP | SNP |
| PCSK9 | INS | SNP |
| TBC1D8 | SNP | SNP |
| ZNF423 | SNP | SNP |
| TOX2 | INS | SNP |
| ZBTB32 | SNP | SNP |
| INSRR | SNP | SNP |
| CFTR | DEL, SNP | SNP |
| GXYLT1 | SNP | SNP |
| NLRP12 | SNP | SNP |
| OVCH1 | SNP | SNP |
| DGKB | SNP | SNP |
| COG7 | SNP | SNP |
| WWC3 | SNP | SNP |
| LRP5 | SNP | SNP |
| MYL1 | DEL | SNP |
| WDR60 | SNP | SNP |
| DSCAM | SNP | SNP |
| NRXN1 | SNP | SNP |
| TANC2 | SNP | SNP |
| TENM2 | SNP | SNP |
| CRB1 | SNP | SNP |
| LAMA1 | SNP | SNP |
| ACVR2A | DEL | SNP |
| SETDB1 | SNP | SNP |
| CCDC110 | SNP | SNP |
| ABL2 | SNP | SNP |
| PROS1 | SNP | SNP |
| FCHO1 | SNP | SNP |
| AKAP9 | SNP | SNP |
| RPTN | SNP | SNP, DEL |
| SRRM4 | SNP | SNP |
| RCOR2 | INS | SNP |
| FCGR2A | SNP | SNP |
| MXRA5 | SNP | SNP |
| AKT1 | SNP | SNP |
| PRRX1 | SNP | SNP |
| PTPRD | SNP | SNP |
| DCLRE1C | SNP | SNP |
| PLK2 | SNP | SNP |
| PIGW | SNP | SNP |
| IGSF3 | SNP | SNP |
| SLC2A4RG | SNP | SNP |
| LCN2 | SNP | SNP |
| RB1 | SNP, DEL, INS | SNP |
| ZNF71 | SNP | SNP |
| GNL3 | SNP | SNP |
| EED | SNP | SNP |
| OR4F17 | SNP | SNP |
| OR51A2 | SNP | SNP |
| CNTNAP3B | SNP | SNP |

**Abbreviations:** TCGA, The Cancer Genome Atlas; WES, whole exon sequencing; DEL, deletion; SNP, single nucleotide polymorphism; INS, insertion.

**Table S3** Luminal and basal/TNBC cell lines and their TTC17 expression levels from the CCLE database.

| Cell line (Luminal) | TTC17 expression  Log_2_ (TPM+1) | Cell line (Basal/TNBC) | TTC17 expression  Log_2_ (TPM+1) |
| --- | --- | --- | --- |
| T47D | 5.912171 | HCC1143 | 5.704042 |
| AU565 | 5.789990 | HCC1187 | 5.692929 |
| UACC812 | 5.762083 | HCC1500 | 5.499846 |
| MDAMB415 | 5.752749 | CAL120 | 5.453188 |
| HCC1428 | 5.701272 | HS578T | 5.393691 |
| SKBR3 | 5.696550 | MDAMB436 | 5.222650 |
| MDAMB175VII | 5.627023 | HCC38 | 5.210623 |
| ZR751 | 5.522621 | HCC1569 | 5.169123 |
| HCC202 | 5.482526 | HCC1937 | 5.059182 |
| HCC2218 | 5.294988 | BT20 | 5.042644 |
| JIMT1 | 5.265287 | HCC1806 | 4.984134 |
| UACC893 | 5.174327 | HCC1954 | 4.976364 |
| ZR7530 | 5.155425 | HCC70 | 4.975447 |
| BT483 | 5.146085 | KPL1 | 4.967630 |
| BT474 | 5.117279 | MDAMB231 | 4.959770 |
| MDAMB134VI | 5.063934 | HCC1599 | 4.866908 |
| CAMA1 | 5.038261 | HCC2157 | 4.687061 |
| EFM192A | 4.956521 | DU4475 | 4.612942 |
| MDAMB361 | 4.942984 | BT549 | 4.607034 |
| HMC18 | 4.912650 | MDAMB157 | 4.593354 |
| HCC1419 | 4.904484 | HCC1395 | 4.581954 |
| MDAMB453 | 4.691534 | CAL148 | 4.297925 |
| MCF7 | 4.620000 | CAL851 | 3.631104 |
| EFM19 | 4.516015 |  |  |

**Abbreviations:** TNBC, triple-negative breast cancer; CCLE, Cancer Cell Line Encyclopedia.
