# Supplementary material for: Loss of TTC17 promotes breast cancer metastasis through RAP1/CDC42 signaling and sensitizes it to rapamycin and paclitaxel
Source: Cell Biosci. 2023 Mar 9;13:50. doi: 10.1186/s13578-023-01004-8 (PMC9996991; doi:10.1186/s13578-023-01004-8)
Supplement: Supplementary file 2 — Additional file 2: Fig. S1 a-p Representative Kaplan–Meier curves of relapse-free survival in patients with breast cancer, stratified by the expression of candidate genes, based on the KM plotter database. The candidate genes were obtained from genome-wide CRISPR screening concurrent with mutation detection in breast cancer tissues (a-h) or differential transcriptional expression in breast cancer using random forest analysis with TCGA data (i-p). Abbreviation: TCGA, the Cancer Genome Atlas. Fig. S2 a-h Kaplan-Meier curves of overall survival in patients with bladder carcinoma (a), pancreatic ductal adenocarcinoma (b), rectum adenocarcinoma (c), stomach adenocarcinoma (d), cervical squamous cell carcinoma (e), kidney renal clear cell carcinoma (f), kidney renal papillary cell carcinoma (g), or pheochromocytoma and paraganglioma (h), stratified by TTC17 expression. Data was obtained from the KM plotter program. Fig. S3 Protein levels of TTC17 in normal and breast cancer tissues based on the CPTAC dataset from the UALCAN portal. Abbreviations: CPTAC, Clinical Proteomic Tumor Analysis Consortium; UALCAN, The University of Alabama at Birmingham Cancer Data Analysis Portal. Fig. S4 Representative images and quantitative analysis of wound healing assays using MDA-MB-231 cells with forced TTC17 expression and control cells. Scale bar, 500 μm. Fig. S5 Graphic display and statistic efficiencies of the colonies formed by MCF7 cells with or without forced TTC17 expression. Fig. S6 Difference in CDC42 expression between BRCA and normal breast specimens using TCGA combined with GTEx data. Abbreviations: BRCA, breast invasive carcinoma; TCGA, The Cancer Genome Atlas; GTEx, Genotype-Tissue Expression. Fig. S7 a-b Western blot analysis of TTC17 and CDC42 expression in MCF7 cells with TTC17 knockdown (a) or overexpression (b) and their counterparts. Fig. S8 Illustration of the role and mechanism of TTC17 on promoting breast cancer metastasis and drug sensitivity via RAP1/CDC42 signal [file 13578_2023_1004_MOESM2_ESM.docx]

**Figure S1**

**
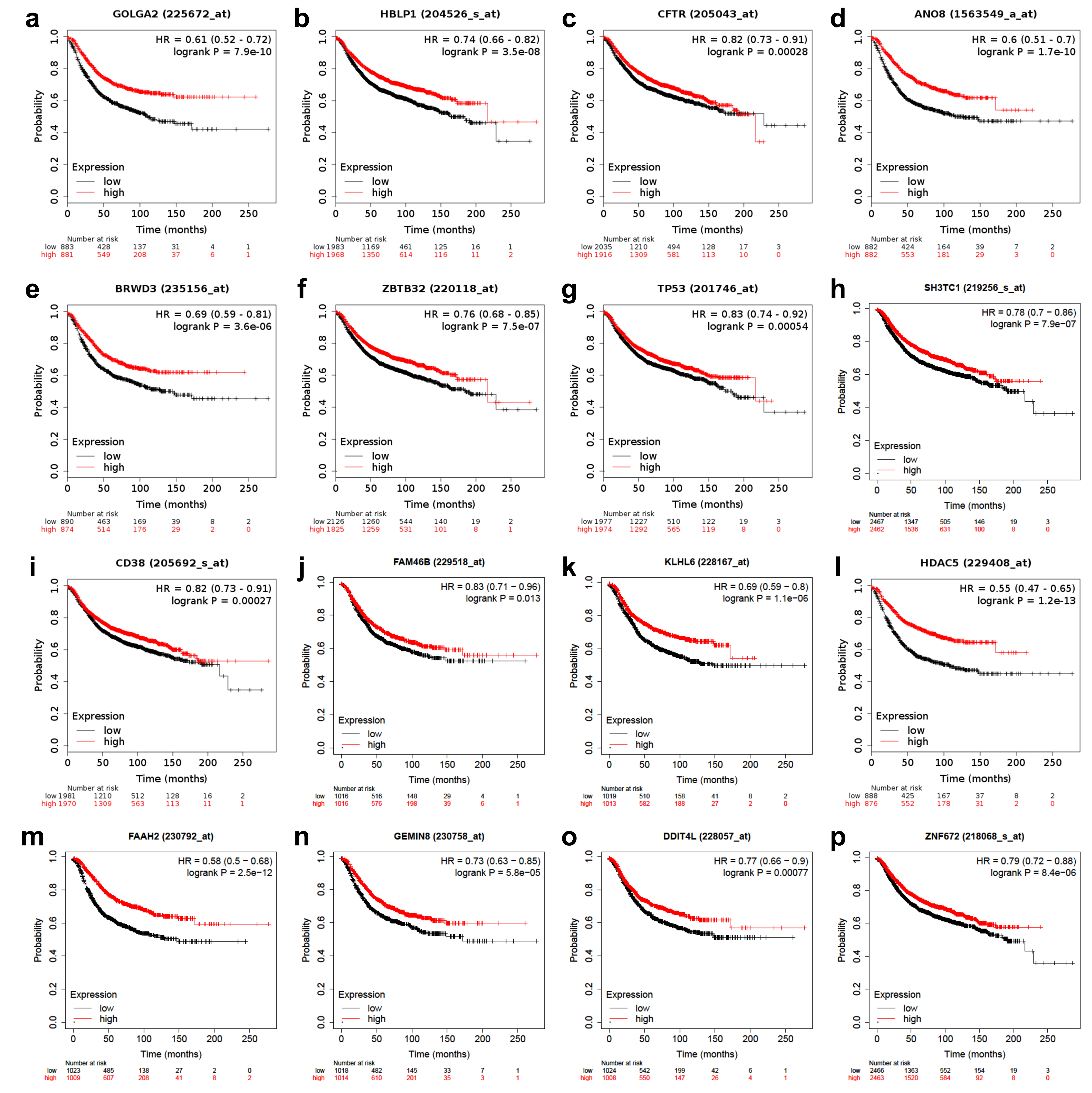
**

**Fig. S1** **a-p** Representative Kaplan–Meier curves of relapse-free survival in patients with breast cancer, stratified by the expression of candidate genes, based on the KM plotter database. The candidate genes were obtained from genome-wide CRISPR screening concurrent with mutation detection in breast cancer tissues **(a-h)** or differential transcriptional expression in breast cancer using random forest analysis with TCGA data **(i-p)**.

**Abbreviation:** TCGA, the Cancer Genome Atlas.

**Supplementary Figure 2**


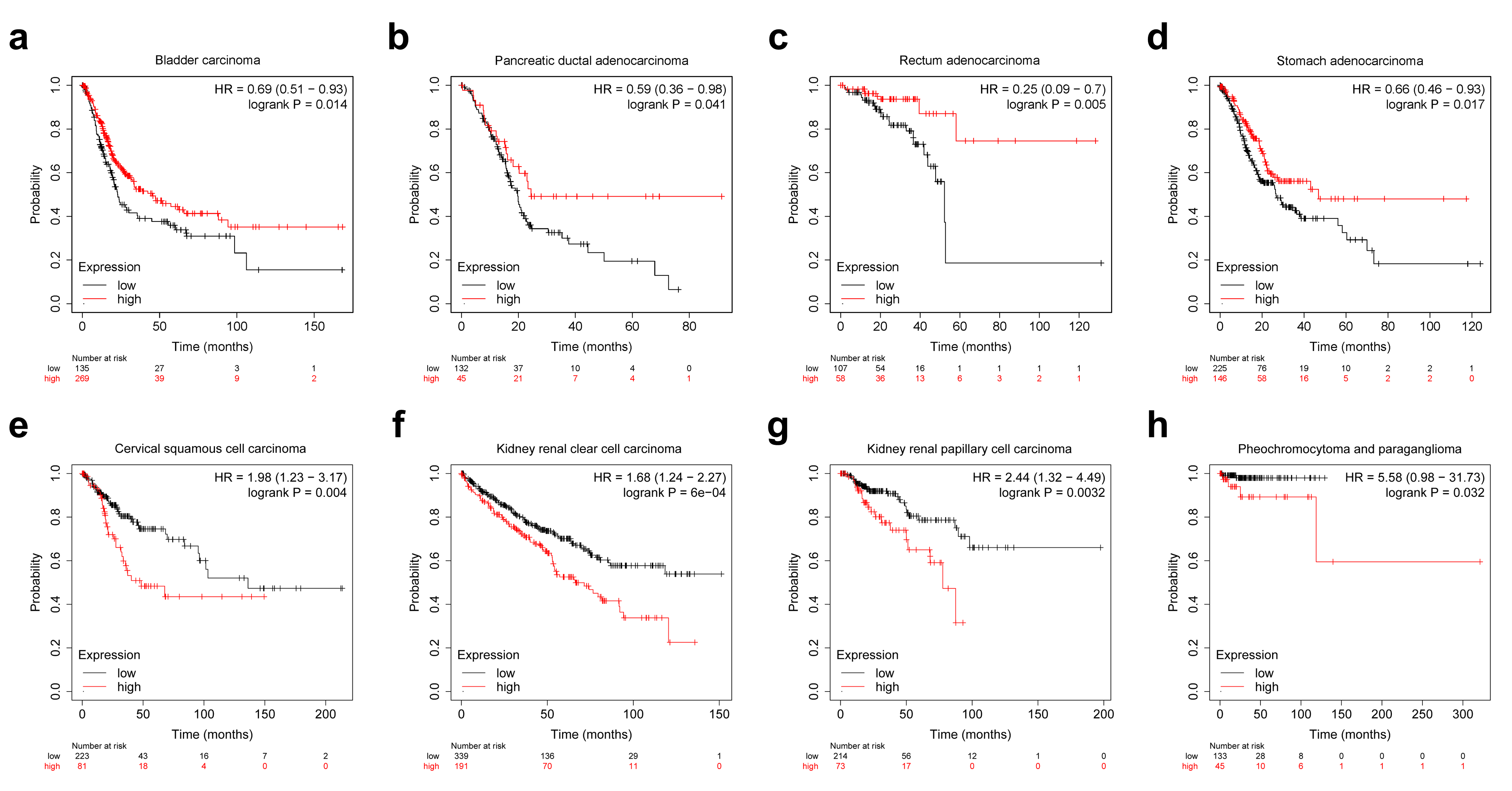


**Fig. S2 a-h** Kaplan‒Meier curves of overall survival in patients with bladder carcinoma (**a**), pancreatic ductal adenocarcinoma (**b**), rectum adenocarcinoma (**c**), stomach adenocarcinoma (**d**), cervical squamous cell carcinoma (**e**), kidney renal clear cell carcinoma (**f**), kidney renal papillary cell carcinoma (**g**), or pheochromocytoma and paraganglioma (**h**), stratified by TTC17 expression. Data was obtained from the KM plotter program.

**Figure S3**


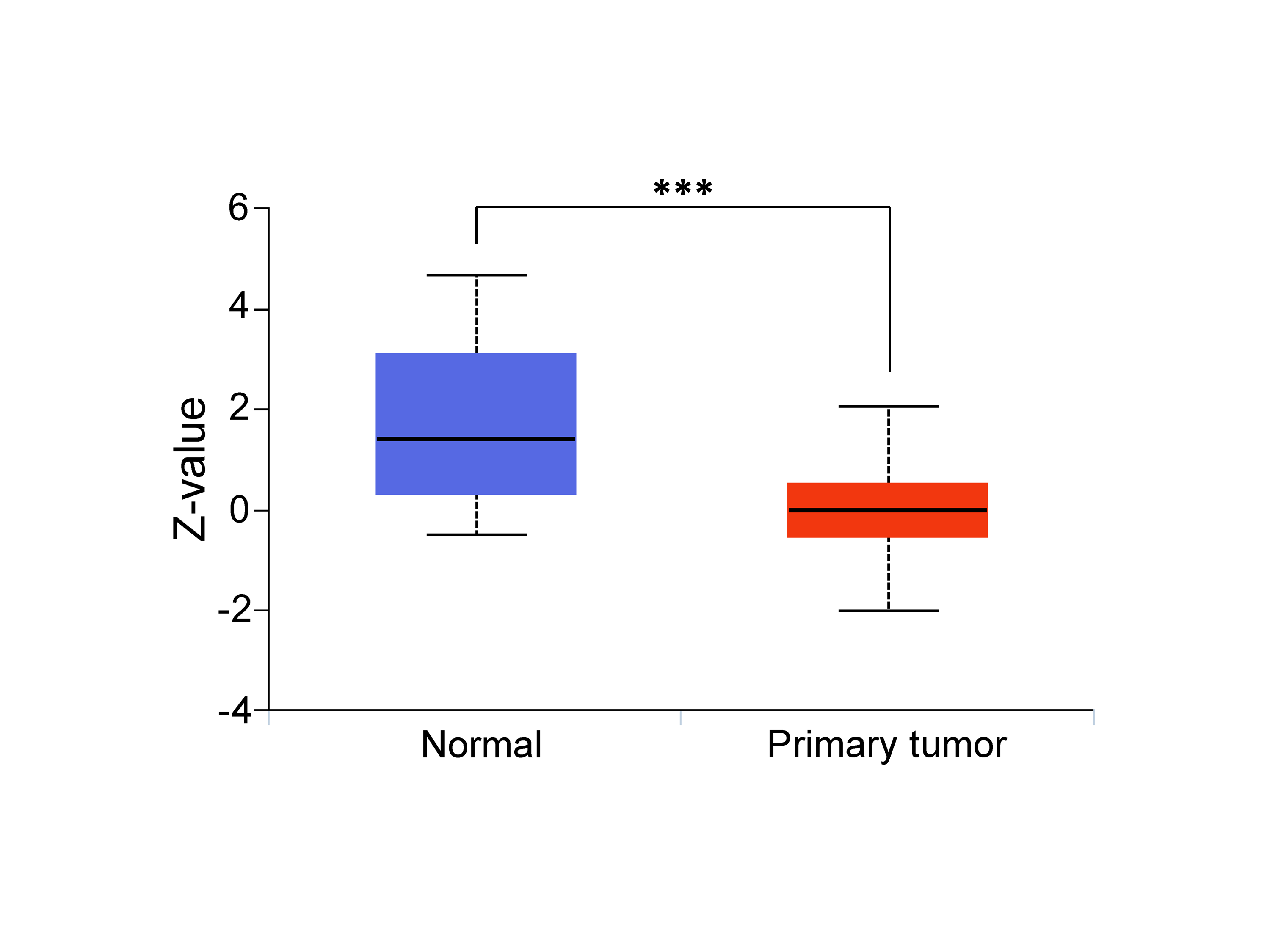


**Fig. S3** Protein levels of TTC17 in normal and breast cancer tissues based on the CPTAC dataset from the UALCAN portal.

**Abbreviations:** CPTAC, Clinical Proteomic Tumor Analysis Consortium; UALCAN, The University of Alabama at Birmingham Cancer Data Analysis Portal.

**Figure S4**


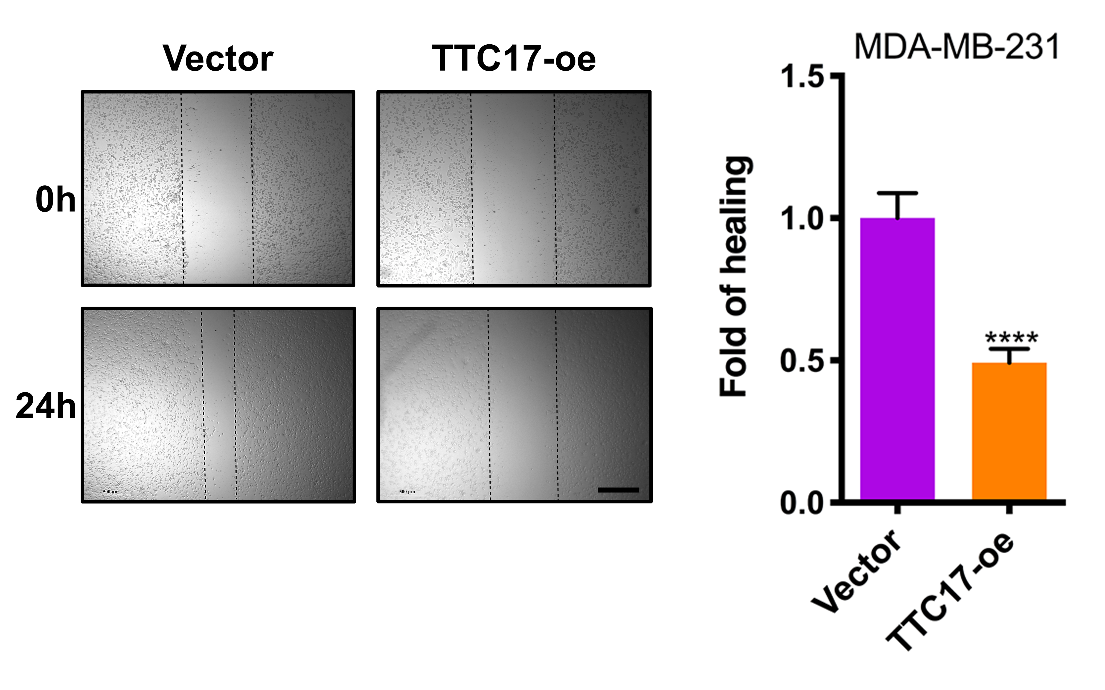


**Fig. S4** Representative images and quantitative analysis of wound healing assays using MDA-MB-231 cells with forced TTC17 expression and control cells. Scale bar, 500 μm.

**Figure S5**


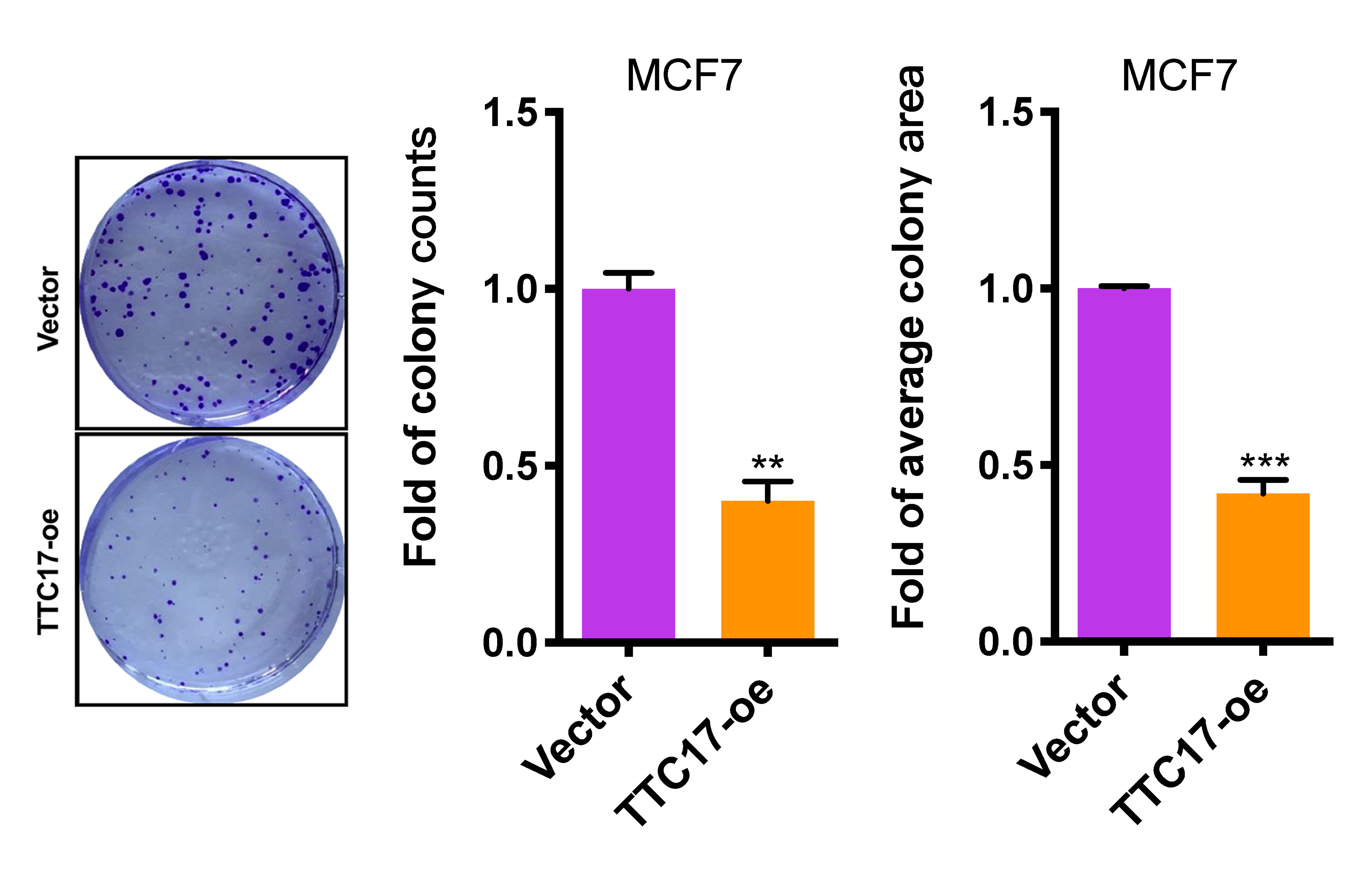


**Fig. S5** Graphic display and statistic efficiencies of the colonies formed by MCF7 cells with or without forced TTC17 expression.

**Figure S6**


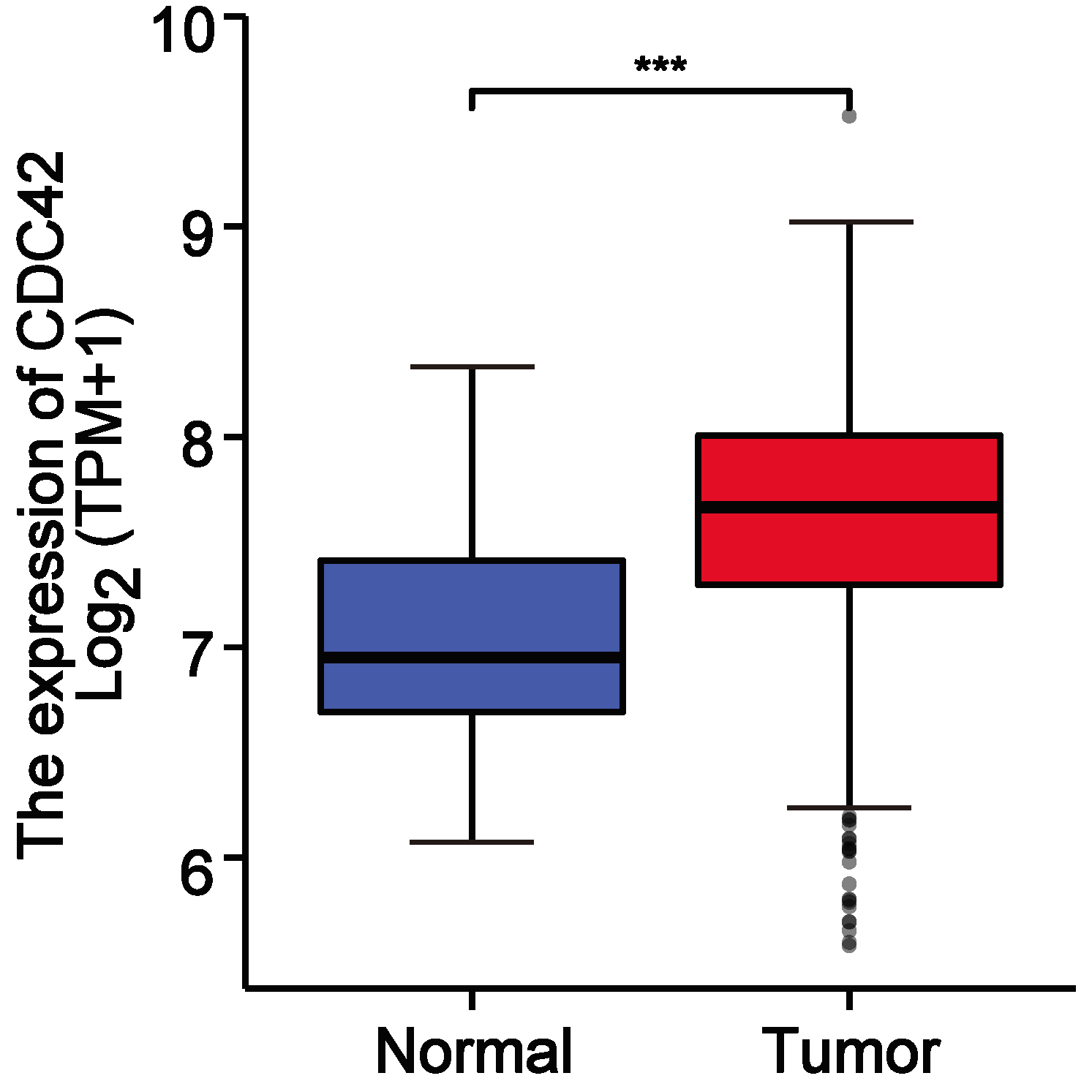


**Fig. S6** Difference in CDC42 expression between BRCA and normal breast specimens using TCGA combined with GTEx data.

**Abbreviations:** BRCA, breast invasive carcinoma; TCGA, The Cancer Genome Atlas; GTEx, Genotype-Tissue Expression.

**Figure S7**


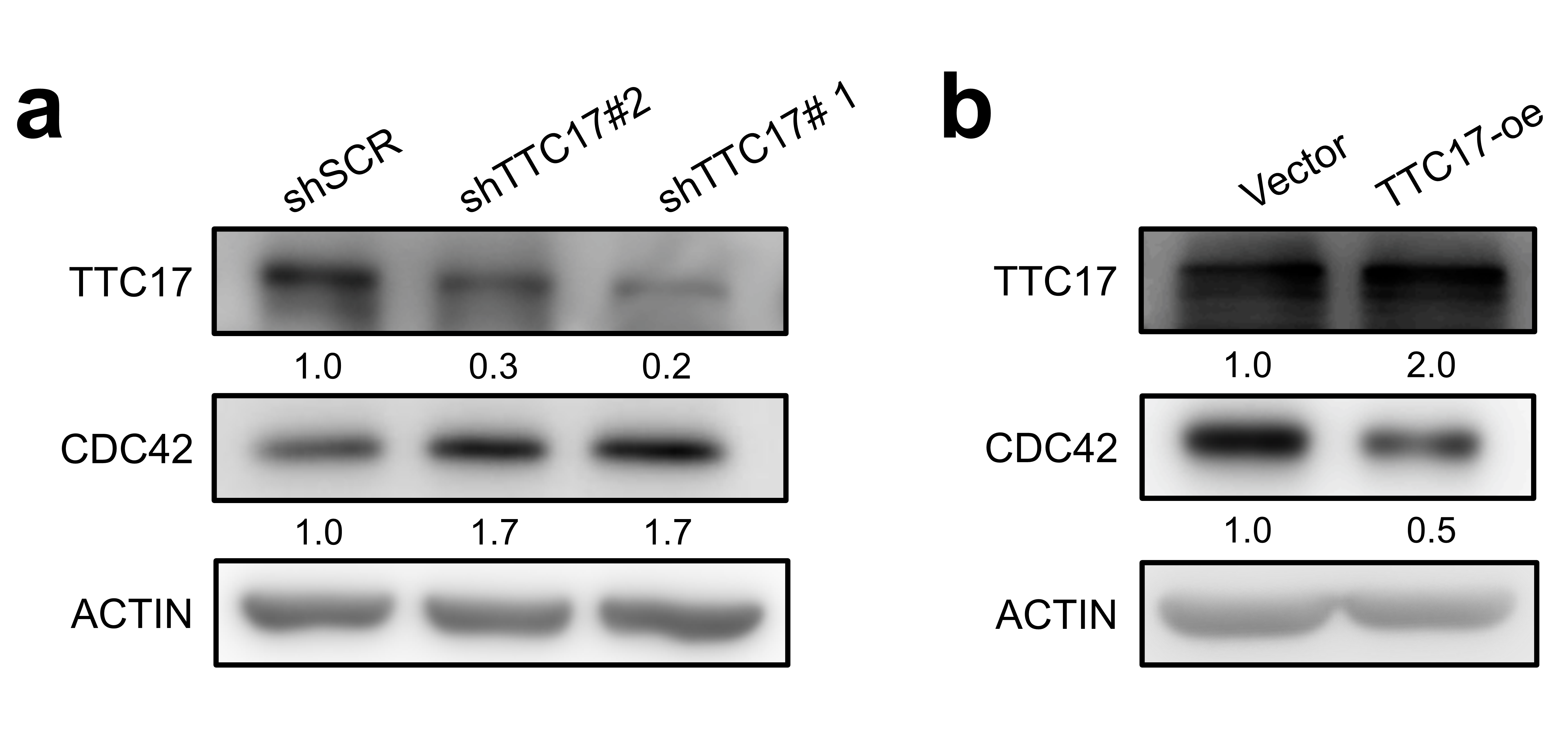


**Fig. S7 a-b** Western blot analysis of TTC17 and CDC42 expression in MCF7 cells with TTC17 knockdown (**a**) or overexpression (**b**) and their counterparts.

**Figure S8**

**
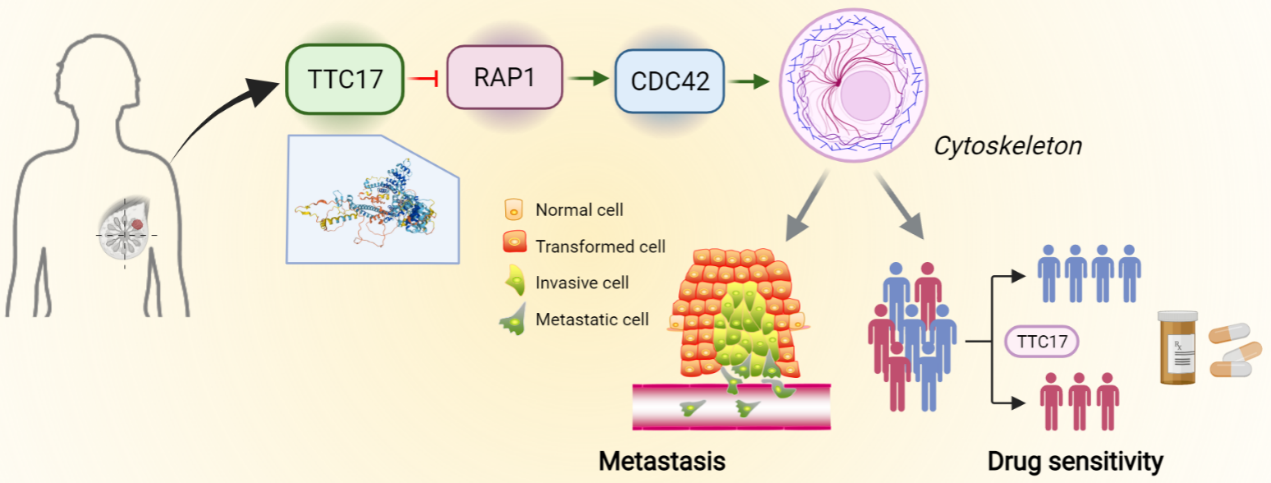
**

**Fig. S8** Illustration of the role and mechanism of TTC17 on promoting breast cancer metastasis and drug sensitivity via RAP1/CDC42 signaling pathway.
